# Data on the time of integration of the human mitochondrial pseudogenes (NUMTs) into the nuclear genome

**DOI:** 10.1016/j.dib.2017.05.024

**Published:** 2017-05-17

**Authors:** Konstantin Gunbin, Leonid Peshkin, Konstantin Popadin, Sofia Annis, Rebecca R. Ackermann, Konstantin Khrapko

**Affiliations:** a Institute of Cytology and Genetics SB RAS and Novosibirsk State University, Russia; b University of Lausanne, Center for Integrative Genomics, Switzerland; c Harvard Medical School, Boston, USA; d Department of Archaeology & Human Evolution Research Institute, University of Cape Town, South Africa; e Northeastern University, Boston, USA

**Keywords:** NUMT, Human evolution, Mitochondrial DNA, Pseudogene, Speciation, Punctuated evolution

## Abstract

The data and methods presented in this article are supplementing the research article "Integration of mtDNA pseudogenes into the nuclear genome coincides with speciation of the human genus. A hypothesis", DOI: 10.1016/j.mito.2016.12.001 (Gunbin et al., 2017) [1]. Mitochondrial DNA is known to get inserted into nuclear DNA to form NUMTs, i.e. nuclear DNA pseudogenes of the mtDNA. We present here the sequences of selected NUMTs, in which time of integration can be determined with sufficient precision. We report their chromosomal positions , their position within the great ape mtDNA phylogeny, and their times of integration into the nuclear genome. The methods used to generate the data and to control their quality are also presented. The dataset is made publicly available to enable critical or extended analyzes.

**Specifications Table**

**Table:** 

Subject area	*Biology*
More specific subject area	*Human Evolution*
Type of data	*text file, table, graph*
How data was acquired	*Sequence data were acquired from the Genbank database using selection criteria for the sequences that allowed reliable determination of the time of NUMT insertion.*
Data format	*FASTA, graphics.*
Experimental factors	*N/A*
Experimental features	*N/A*
Data source location	*N/A*
Data accessibility	*The data are available with this article*

**Value of the data**

– *The data presents* the selected NUMTs in which time of integration can be determined with sufficient precision, their sequences and their chromosomal positions in the human nuclear genome.
– *The data presents* the position of the selected NUMTs within the great ape mtDNA phylogeny (Fig. S4 - available in the Supplementary Material), their times of integration into the nuclear genome.
– The dataset enables critical and extended analyzes of the human evolution features revealed by the analysis of these NUMTs [1].

## 1 Data

**Fig. S1:**
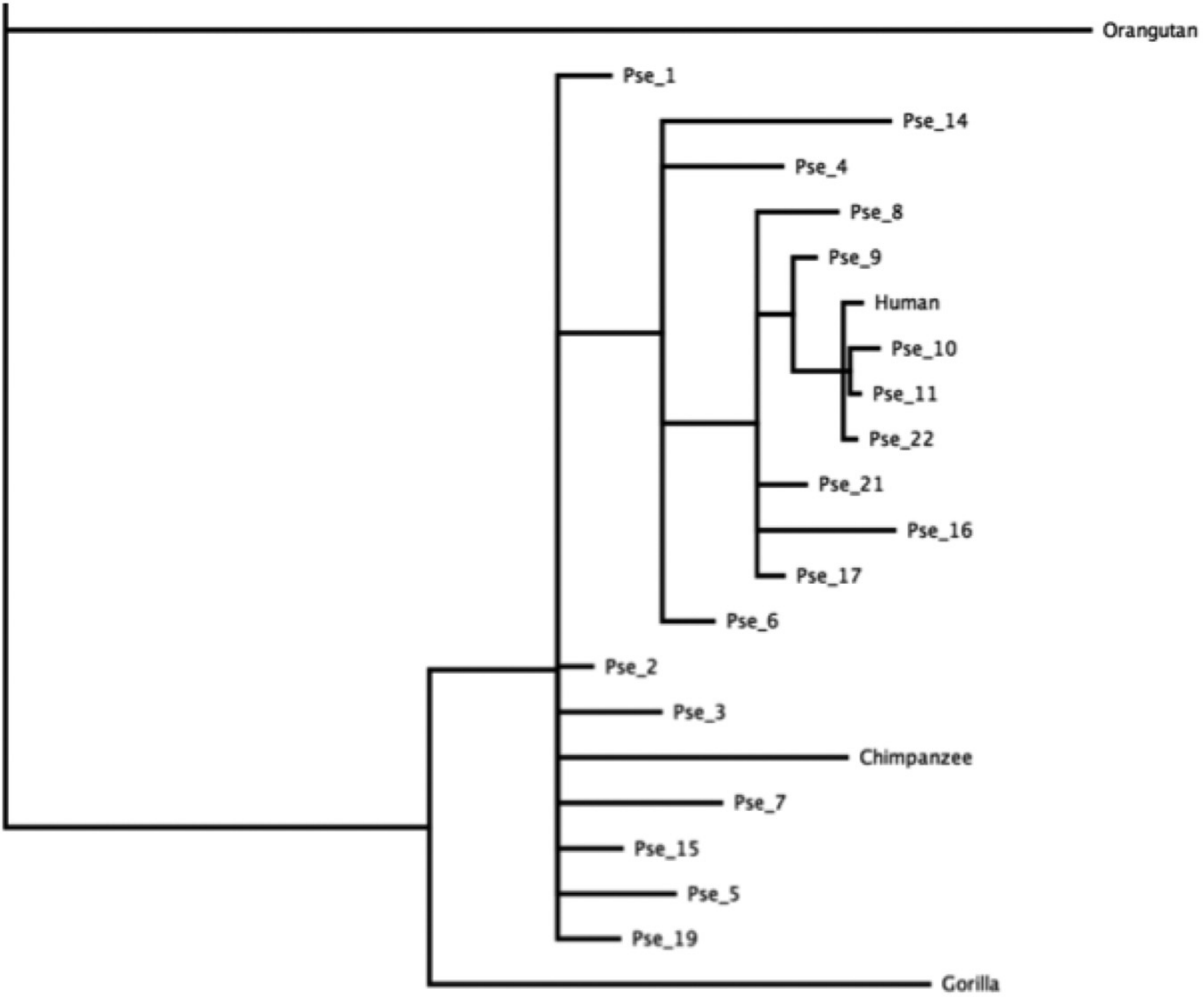
**18 Selected NUMTs are not products of post-insertion duplication.** Joint Bayesian phylogenic tree of the entire set of 18 selected NUMTs and the 5 higher ape mtDNA sequences. The Gibbon used as outgroup is not shown. Note that none of the NUMTs have any stems in common other than those shared with at least one mtDNA sequence. This implies that among the selected 18 NUMTs there were no duplications after the insertion into the nuclear genome.

**Fig. S2:**
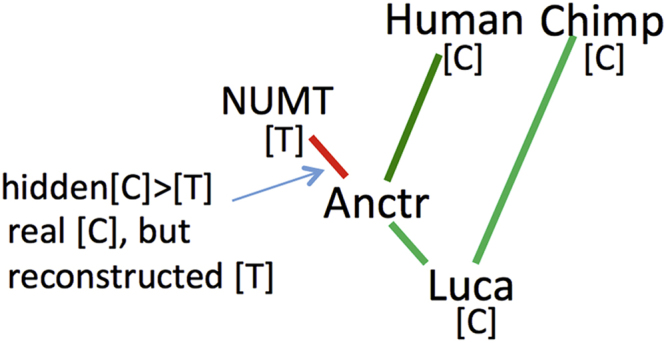
**Example of a “hidden” substitution.** “Anctr” (for Ancestor), the coalescence point of the NUMT branch and the human mitochondrial lineage (“Human”). “Luca” – coalescence point of the human and the Chimpanzee (Chimp) lineages. A “hidden” substitution arises, for example, when a position in the reconstructed interior node Anctr is determined in error (T instead of the real C). Then the true C>T substitution in the NUMT branch becomes “hidden”. Hidden substitution can be revealed by manual analysis of reconstructed ancestral sequences. The reconstruction of ancestral sequences was made by marginal algorithm based on maximum likelihood estimation of parameters.

**Fig. S3:**
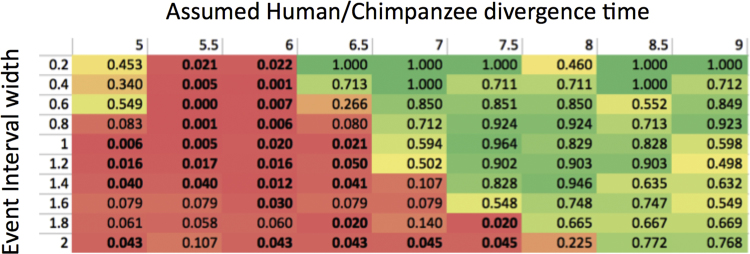
Probability of equally or more extreme (more clustered) distribution of random data points as compared to the distribution of the actual NUMTs’ insertion times. (See Section 2.6).

The data presented in this article consists of the sequences of the selected NUMTs, which were chosen for being suitable for high resolution phylogenic analysis (Supplementary Material, Appendix A). Also presented are their chromosomal positions and times of integration (Table S1 - Supplementary Material, Appendix A), the trees representing the relation of the selected NUMTSs to the great ape mtDNA phylogeny are presented in Fig. S4 (Supplementary Material, Appendix A). The interrelationships between the NUMTs themselves are presented in Fig. S1. The times of integration of the selected NUMTs into the nuclear genome are presented in Table S1. Methods pertaining to the sources, generation and verification of the data are described in Materials and methods, and Fig. S2 and S3.

## 2 Materials and methods

### 2.1 Data sources (S.1)

78 nuclear mitochondrial DNAs (NUMTs) were obtained from Tsuji et al. [2] (hg18-numts.tsv file). We included NUMTs that were clustered with hominid branches of the primate tree (i.e., f, e, d branches in Fig. S1 of Supplementary Material of Tsuji et al. [2]. We used all 78 pseudogenes, including those flagged as “possible duplications”. 23 NUMTs were obtained from Dayama et al. [3] (sequenced/mapped NUMTs only, i.e. 23 out of 141 detected). Thus overall, we started with 101 human NUMT sequences.

### 2.2 Data selection/filtration (S.2)

#### 2.2.1 Time interval 0–6 Ma (S.2a)

The focus of this study is the timing of NUMT insertion into the human nuclear DNA lineage after the human-chimpanzee divergence. In accordance with this, we subjected the starting set of NUMTs to a two-step selection/filtering process. We therefore first filtered out NUMTs that were certainly outside the target evolutionary period 0 to 6 Ma (see discussion of the uncertainty of this range in Section 2.6). For that purpose, each NUMT sequence was aligned with the reference *Homo sapiens sapiens* mtDNA sequence (GenBank ID: NC_012920.1). The divergence was determined from pairwise sequence alignments via MAFFT v. 6.847b [4] (default aligning options) using single or concatenated *H. s. s.* mtDNA. All pairwise sequence alignments were scored by distmat program (option: -nucmethod 0) from the EMBOSS v. 6.3.1 package [5]. NUMT sequences that were divergent from the human mtDNA sequence by more than 10% of positions (according to MAFFT alignment) were filtered out. This criterion is conservative because such NUMTs are safely out of our target evolutionary period, as chimpanzee mtDNA is divergent from the human sequence by a maximum of 8% by this approach.

To make sure that the sequences that are specifically problematic for MAFFT were not discarded because of being misaligned, we *additionally* performed search using the blastn program (default options) from the BLAST v. 2.2.26+ package [6]. BLAST helps to deal with long gaps, which are poorly handled by MAFFT. After mapping the blastn NUMTs onto human mtDNA, we discarded poorly mapped NUMTs using the log10 E-value score outlier criterion (score boundary is Q3+0.5*(Q3-Q1), where Q1 and Q3 are 25th and 75th percentiles of log10 E-value scores for all NUMTs, the constant 0.5 was selected by manual inspection of gap width in E-value scores. The constant of 0.5 provides a clear division between two group of scores – high, 85–100% of similarity, and low, less than 65% of similarity). The use of these criteria resulted in the set of 52 NUMTs.

#### 2.2.2 Topology criterion (S.2b)

Second, we filtered out NUMTs associated with incorrect tree topology.

The rationale for this criterion is as follows. As described in Section 2.3 below, a multiple sequence alignment with a cassette of mitochondrial genomes of higher hominids (human, chimpanzee, gorilla) was used to reconstruct phylogeny of each NUMT, determine branch lengths, and eventually estimate the time of NUMT insertion. This estimate critically depends on the correct reconstruction of the human/chimpanzee/gorilla phylogenic tree: if tree topology is incorrect or unstable, the estimates of NUMT insertion time become meaningless, as the time of insertion is determined based on the topology and the branch lengths of this tree. Of note, to avoid mutation rate biases (mutation rates vary between different regions of mtDNA), the alignment for each NUMT with the cassette of mtDNA sequences must be constructed using exclusively the mtDNA sub-fragment homologous to the NUMT sequence. Thus the sequences of the mitochondrial genomes outside the NUMT boundaries were deleted from the alignment. However, some of the NUMT sequences were so short that the corresponding mtDNA sequences were not sufficient to robustly reconstruct the correct phylogenic tree. NUMTs were not considered for further analysis if the support of the correct topology in the species (human/chimpanzee/gorilla) tree (constructed as described in Section 2.3) was below 70%, which indicated a highly unstable topology.

The combined selection/filtration procedures (sections *a* and *b*) resulted in 18 NUMTs (see Table S1 Supplementary Material, *Appendix A*)

#### 2.2.3 Independence of insertions (S.2c)

We then tested whether any of the selected NUMTs were more closely related to each other than they were to the mtDNA , which would imply that they could have been derived from a single ancestral NUMT after it had significantly diverged from mtDNA. The importance of this test is that such two NUMTs should not be counted as independent insertion events in our statistical analysis.

To test for relationships between the 18 selected NUMTs, we constructed a joint phylogeny of the entire set of pseudogenes together with the full-length human, chimpanzee, gorilla mtDNA sequences plus orangutan and gibbon mtDNA as outgroups. The rationale of this analysis is that if two pseudogenes were derived from a single ancestral pseudogene, in such a tree, they would have a common stem representing evolution of such ancestral pseudogene.

Note that in this case (unlike in the trees described in Section 2.3 and shown in Fig. S4), we did not exclude any sections of mtDNA from the alignment. This is because the selected 18 NUMTs collectively cover almost the entire mtDNA, so essentially no segment could be excluded from a joint alignment/tree. That is the entire mitochondrial genomes and the NUMT sequences were used in a single alignment without any trimming. The alignment was created using MAFFT web-server [4] with default parameters. The tree was then constructed using Mr.Bayes v. 3.2.6 embedded in the Geneious 10 software suite [7] using the GTR substitution matrix. We note that this joint tree should not be used for branch length estimate s, as we have necessarily violated all the special precautions that we otherwise took to estimate the unbiased insertion times (Section 2.3). In this tree, different regions of mtDNA are pooled together, no correction is made for the length differences between pseudogenes, and the excessive complexity of the tree undoubtedly impedes the ability of the algorithm to assign correct branch lengths. We caution therefore that while this tree serves its purpose to illustrate the lack of significant kin associations between pseudogenes, it should not be used for more delicate task of determining insertion times.

In conclusion, as seen from the tree (Fig. S1) there is very little apparent relation between different pseudogenes beyond their relation to mtDNA, so the scenario of NUMT insertion with subsequent duplication after substantial time period appears unlikely for any of our selected 18 NUMTs. Immediate duplications after insertion are not excluded by this analysis but would be considered independent mutations within the same chronological epoch, so counting them as independent events for the purpose of testing of our hypothesis (Section 2.6) is fully justified.

### 2.3 Determining the branch lengths (S.3)

To determine branch lengths, we first align each NUMTs with a cassette of sequences comprising 81 mtDNA of higher primates. These multiple alignments include 72 mtDNA sequences published in [8] (30 gorilla sequences, 33 chimpanzee sequences and 9 human sequences), plus 8 mtDNA sequences from ancient humans (GenBank IDs: KF683087.1, KF982693.1, NC_013993.1, KC879692.1, NC_011137.1, KJ533545.1, NC_023100.1, KJ533544.1) and one consensus mtDNA of extant human (GenBank ID: NC_012920.1). Alignments were constructed using sate v. 2.2.7 program [9] (default options). After that, the parts of alignment outside the NUMT boundaries were deleted .

For each of the NUMTs we constructed a set of 200 of their joint phylogenetic trees with the 81 primate mtDNA sequences. This was done using raxML v. 8.1.21 [10] (options: -f d -m GTRGAMMA) and in-house 37% [11] diversity-awarded jackknife procedure. In our analyses, we used GTR model of nucleotide substitutions because we have determined, using the jModelTest v. 2 [12] that this model is the best-fit model of nucleotide substitution as determined by all information criteria implemented in jModelTest v. 2 (AIC, AICc, BIC and DT) for all samples containing NUMTs and 81 primate mtDNA sequences. The diversity-awarded jackknife is an in-house modification of the standard jackknife procedure, wherein the probability of the deletion of an alignment column (37% of alignment columns were deleted) depends on the “diversity” of the corresponding nucleotide position calculated as in [13]. The basic idea of the diversity-awarded jackknife procedure is to weigh nucleotide positions in favor of those with evolutionarily fixed nucleotides (functionally important) and against highly variable positions (nearly neutral). The less conserved a position is, the higher the position “diversity” and, therefore, the higher the probability of deletion during the jackknife procedure. Such a procedure can be well suited for discriminating a functionally important phylogenetic signal from a neutral one [13]. This procedure is critical because neutrally evolved positions usually occurred in several alternative states in one clade, which in turn leads to artificial branch clustering especially when the number of evolutionary changes is too small to be used in standard random jackknife. The consensus trees were obtained using sumtrees.py program from DendroPy library v. 3.3.1 [14] (options: --edges mean-length -f0.25). At this point we removed the NUMTs with incorrect or unstable topology: all NUMTs where the consensus tree had incorrect topology or the support for human or chimpanzee branch was lower than 70% were removed from further analysis (see also Section 2.3). Optimal phylogenies for the remaining 18 NUMTs are shown in Fig. S4 (Supplement). The 18 alignments were further manually inspected for possible algorithm errors. Indeed, a gap in the NUMT Pse_8 sequence (as compared to the mtDNA) resulted in two alternative alignments of this NUMT and two branch lengths. We selected the alignment with the with shorter gap because it corresponds to case with the lowest (1.3 times lower) number of parsimonious substitutions onto the NUMT branch of the phylogenetic tree. The 200 jackknife trees created for each NUMT were further used to assess the *variance of our insertion time estimates* (Section 2.5).

This approach yielded branch lengths in “substitutions per nucleotide”, where every substitution is weighted by the substitution rates in the corresponding GTR matrix. Note that this measure is different from the “number of mutations per branch” as determined in Section 2.4 using ancestral state reconstruction, which reflects the actual number of mutations according to our best estimate.

### 2.4 Determining the fraction of pseudogenic substitutions on the NUMT branches (S.4)

In order to determine the fraction of pseudogenic substitutions on a NUMT tree branch we first reconstructed the ancestral sequences of each interior node of the phylogenic tree. This allowed us to *assign* mutations to specific tree branches. Second, based on the comparison of the types of changes that occurred on a NUMT branch to the types of those that occurred on the mitochondrial branches, we calculated the fraction of pseudogenic substitutions on that NUMT branch, as described in more detail below.

*Reconstructions of ancestral sequences* were made in each interior node of the best-scored binary (non-polytomic) NUMT/mtDNA hybrid phylogenetic tree chosen from 50 alternative trees reconstructed using PHyML v. 20120412 program [15] (options: -d nt -b 0 -m GTR -f e -t e -v e -a e -o tlr -c 6 --n_rand_starts 50 -s SPR --print_site_lnl) that were ranked by AU-statistics using consel v. 0.20 package [16] (catpv program option: -s 9). In this procedure, we used full-length alignments (jackknifed alignments were used for the topology robustness check). We intentionally used the AU test and the consel package because it allows us to take into account the differences in evolution for different sites in alignment, which is especially important when (1) the mitochondrial portion of NUMT sequence evolution is often associated with selection and, (2) the NUMT insertion in the nuclear genome is not accurate, which may lead to the accelerated atypical evolution of nucleotides located at the NUMT flanks. 50 alternative trees were generated using purely random NJ.

The reconstruction of ancestral nucleotides was made by RaxML 8.1.21 [10] (options: -m GTRGAMMA -f A -t), while the reconstruction of ancestral indels was made by prank 121218 [17] (options: -DNA -keep -uselogs). RaxML 8.1.21 used a *marginal algorithm* for ancestral sequence reconstruction based on maximum likelihood estimation of parameters. We resolved not to use parsimony methods, especially for parsimony-informative sites, because parsimony methods have been demonstrated to be significantly inferior to the maximum likelihood approach [18]. Additionally, we determined the positions and types of “hidden**”** substitutions which have likely occurred in the NUMT lineage but appear to be located on neighbor branches (Fig. S2). These two procedures (estimation of ancestral states and checking for “hidden” substitutions) allow us to estimate accurately the set of substitutions that had occurred on NUMT branch.

“Hidden" substitutions were determined by manual analysis of reconstructed ancestral sequences. We assumed, that if all terminal branches in the NUMT-containing clade and its ancestors had in a evolutionary conservative site the nucleotide C (for example) and only the NUMT branch had the nucleotide T, then it was highly unlikely that evolution had the C>T>C path (with a secondary substitution) in the inner tree stem. Thus we accepted the more ”locally parsimonious” hypothesis that the substitution occurred on the NUMT terminal branch.

To determine the fraction of *specifically pseudogenic substitutions* (i.e. substitutions that happened *after* the integration into the nuclear genome) in a given NUMT branch (i.e. branch leading to a NUMT, see Fig. 1 of **[1]**), we first determined GERP scores [19] of mtDNA nucleotide positions in the regions of the mitochondrial genome homologous to each NUMT. Note that because GERP scores are calculated for a position in the genome, all possible point mutations at a given position are assigned the same score. GERP scores varied between −9.75, (highest degree of sequence conservation), and 4.87, (highest degree of variability). The scores were binned into two equally spaced categories: the “conserved” (−9.75 to −2.44) and the “variable” (−2.44 to 4.87), and for each branch of each tree the fraction of “variable” mutations was calculated.

Then, for each of 18 phylogenic trees corresponding to the 18 selected NUMTs (Fig. S4), we considered 3 types of mutations: 1.) NUMT mutations (“_numt_”), i.e. those mutations that were mapped to the NUMT branch based on reconstructed ancestral sequences, 2.) Mitochondrial mutations (“_mito_”), mapped to the human mitochondrial branch, and 3.) The “simulated pseudogenic mutations” (“_psd_”), i.e., random sets of mutations generated 500 times on the corresponding genome fragment. For each simulated set, the number of randomly selected positions was equal to the number of changes observed on the corresponding NUMT branch. Then, for each of the 18 trees, we calculated the *fractions* of “variable” (high GERP) mutations: F_numt_ (for mutations of the NUMT branch), F_mito_ (for the mutations of the human mitochondrial branch) and F_psd_ (average over the 500 sets of randomly generated “simulated pseudogenic” mutations). The fraction of “variable” mutations was high on the mitochondrial branch because these mutations are under selection constraints and “conserved” mutations are mostly not allowed. The fraction of “variable” mutations among the “simulated pseudogenic” mutations was low, because these mutations are not under selection, and the mutations of the NUMT branch (F_numt_) was intermediate, because mutations on the NUMT branch are a mixture of mitochondrial mutations and pseudogenic mutations. The fraction of mitochondrial substitutions on the NUMT branch was then calculated following a simple linear model, i.e., as 1−(F_mito_−F_numt_)/(F_mito_−F_psd_).

### 2.5 Determining of the time of NUMT insertion into the nuclear genome (S.5)

To estimate pseudogenization times, we multiplied the entire NUMT branch length (expressed as % divergence, as determined by the max. likelihood procedure described in Section 2.3) by the fraction of mitochondrial substitutions in that branch (Section 2.4). In this way we determined the length of the mitochondrial DNA segment of the NUMT branch (see Fig. 1 of [1]). We then added the length (as % divergence) of the branch connecting the NUMT branching point with the human-chimpanzee coalescence point. After that we multiplied the resulting number by 6My and divided by the length of the human branch (from its divergence with chimpanzee). This latter transformation was done to convert % divergence into Ma, using Human branch as the standard (human branch was chosen because it is the closest (to the pseudogene) branch of the ape tree). In this way we determined the chronological length of the NUMT branch from the point of human/chimp divergence (6 Ma) to the point of integration into the nuclear genome. Thus to determine the time of integration in Ma (Million years ago) we had to subtract this chronological length from 6 Ma (Fig. 1 of **[1]**).

Confidence intervals of NUMT insertion times (as presented in Fig. 2 of **[1]****)** were determined by repeating the above calculation for 200 trees constructed using the diversity-awarded jackknife (Section 2.3). The mean, median, 25th and 75th percentiles and the variance of the resulting values were then calculated for each of 18 sets (18 NUMTs) of 200 values.

### 2.6 Checking the probability of clusterization of pseudogenization times by random chance (S.6)

To estimate the probability of clusterization of NUMT insertion times in a certain time interval by chance we generated 10^6^ 18–point random data sets drawn from the uniform distribution between 0 and 6 Ma. Then we calculated the fraction (i.e., probability of occurrence) of those 18-point sets that were “as highly clustered or more extremely clustered than the observed distribution of NUMT insertion times” (i.e., distribution in Fig. 2 of **[1]**). The “as clustered or more extreme” set was defined as a set where the number of time points that landed within the interval of the major climate change 2.5-2.9 Ma was equal to or higher than 6 (6 is the number of points in this interval in the distribution NUMT insertion times). The corresponding probability is 0.0006, indicating that it is highly unlikely that the distribution in Fig. 2 of **[1]** has arisen by chance.

This impressively low probability, however, might have been expected to strongly depend on two parameters.

First is the size of the interval used in the calculations. Considering the small number of data points, we might have been simply lucky that the time interval associated with climatic change happened to fit 6 of our data points. We therefore confirmed that this result was not a case of “*p-*value picking” by repeating these estimates with different widths of intervals centered at 2.8 Ma (Fig. S3).

Second, timing of the NUMT insertion points in our model depends linearly on the assumed timing of the Human / Chimpanzee divergence. This latter timing is subject to considerable uncertainty. For example, Prado Martinez [8] reports ~5.5 Ma, different chapters of the most recent authoritative book by Tibayrenc and Ayala (2016) reports ~6 Ma and ~6.6 Ma (Chapters 2 and 8) [20], and Langergraber et al. [21] −7 to 8 Ma. We are not in a position to participate in this complicated discussion, though discrepancies are likely to be caused, at least in part, by the choice of the parts of the genome that are used to determine divergence time. Of note, mtDNA divergence is expected, at least in theory, to most closely follow the evolution of the actual populations, as it is less subject to incomplete lineage sorting because of a 4-fold smaller effective population size.

To address, to the greatest possible extent, the divergence time uncertainty and the interval width issue, we explored the full 2-D array of p-values associated with the hypothesis (“a higher rate of NUMT insertion into nuclear genome was accelerated around 2,8Ma”), which were calculated for different divergence times and the different the event-enclosing interval widths.

To do so, we calculated insertion times for each of 18 NUMTs under assumption of different divergence times (5, 5.5, …, 9 Ma). This was done by appropriately scaling data of Fig. 2 [1], and counting, for each divergence time, the number of NUMT insertion times that were within each of intervals (0.2 to 2Ma) around the 2.8Ma time point (which of course was not scaled). Then we repeated, for each divergence time, 10^6^ randomizations of 18 points within appropriately scaled time interval. For each randomization (for each combination of divergence time and interval width) we counted the number of data points that landed within tha1t interval. Finally we calculated the fraction of randomizations where equal or larger number of data points landed within the interval, compared to what was observed for 18 NUMT insertion times calculated at the same parameters. These fractions of randomizations were used as proxy of the p-values.

The results of this analysis are shown in Fig. S3. As seen in Fig. S3, *p*-value stayed well below the critical 0.05 value within a wide range of interval widths and human/chimp divergence times.At the same time, these results put limitations on our conclusions. For example, if divergence time between humans and chimpanzees were as ancient as 8Ma, then our data are not sufficient to statistically support temporal association of accelerated NUMT insertion into nuclear DNA with the emergence of Homo and/or climatic change 2.8 Ma.

## Appendix A Supplementary material

Fig. S4. Consensus trees of 18 selected NUMTs.

.

Table S1. Descriptions of the 18 selected NUMTs

## Footnotes

Appendix A Supplementary data associated with this article can be found in the online version at http://dx.doi.org/10.1016/j.dib.2017.05.024.
