# Supplementary material for: Data on the time of integration of the human mitochondrial pseudogenes (NUMTs) into the nuclear genome
Source: Data Brief. 2017 May 17;13:536–44. doi: 10.1016/j.dib.2017.05.024 (PMC5491396; doi:10.1016/j.dib.2017.05.024)
Supplement: Supplementary file 1 — Supplementary material [file mmc1.docx]

The authors declare no conflict of interest.

On the behalf of all authors,

Konstantin Khrapko, PhD,

Professor

Department of Biology

Northeastern University

Boston MA 02115
