# Supplementary figures and images for: Data on the time of integration of the human mitochondrial pseudogenes (NUMTs) into the nuclear genome
Source: Data Brief. 2017 May 17;13:536–44. doi: 10.1016/j.dib.2017.05.024 (PMC5491396; doi:10.1016/j.dib.2017.05.024)

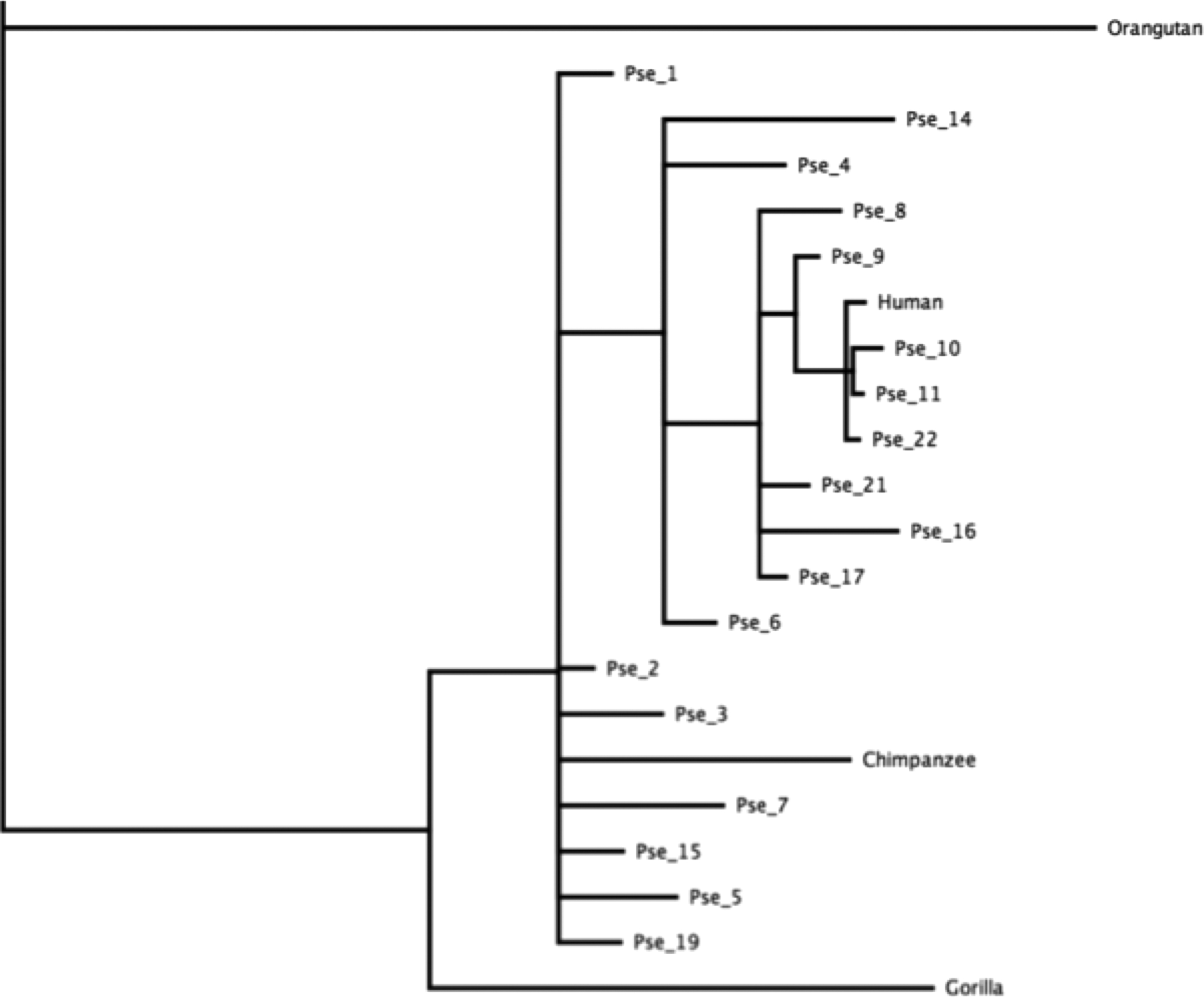

Supplement: Supplementary file 2 — Fig. S4. Consensus trees of 18 selected NUMTs. [file mmc2.jpg]

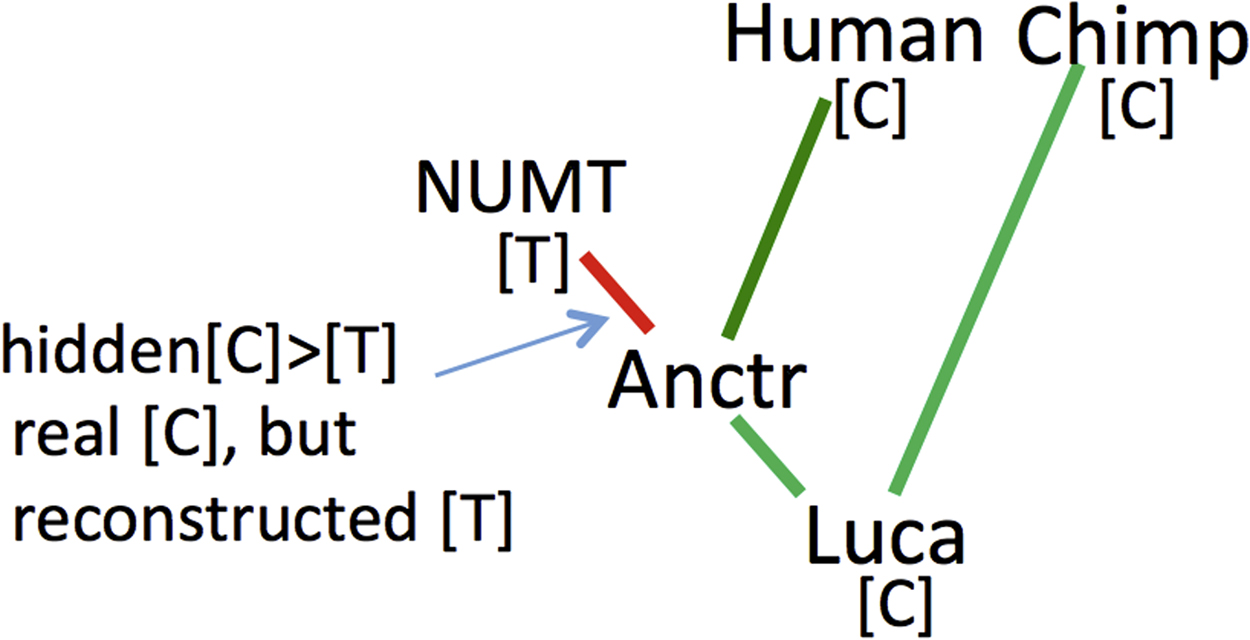

Supplement: Supplementary file 3 — Table S1. Descriptions of the 18 selected NUMTs [file mmc3.jpg]

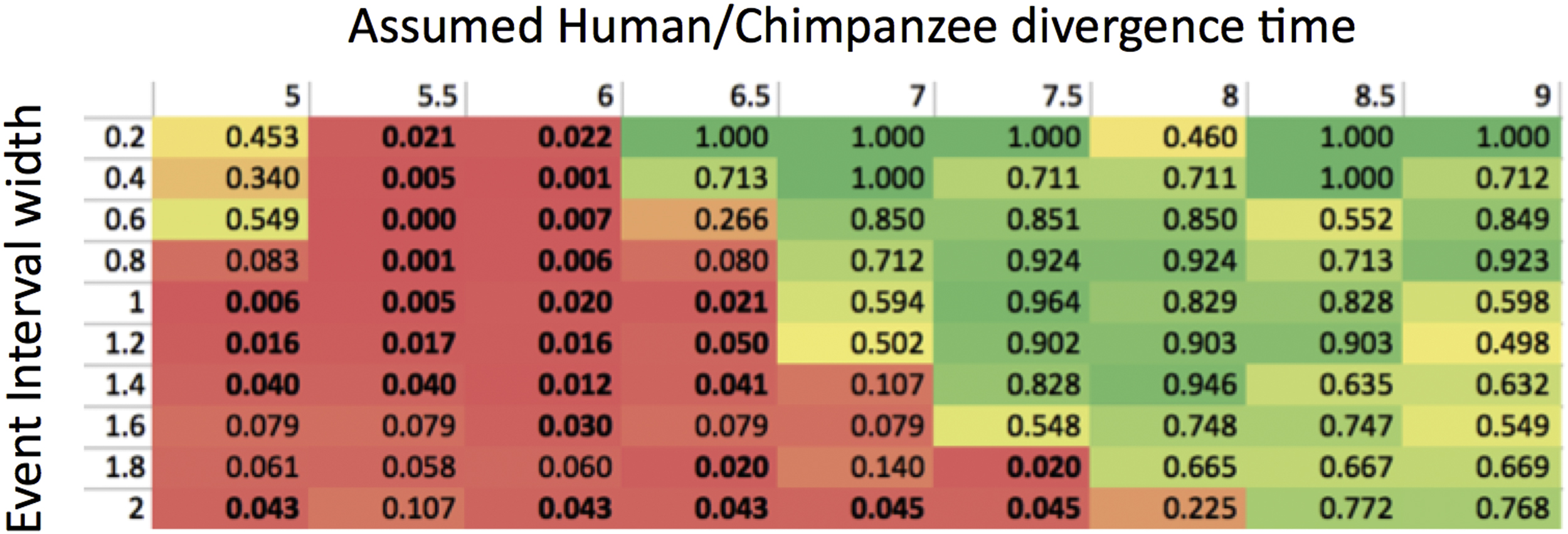

Supplement: Supplementary file 4 — Table S1. Descriptions of the 18 selected NUMTs [file mmc4.jpg]
