# Supplementary material for: Data on the time of integration of the human mitochondrial pseudogenes (NUMTs) into the nuclear genome
Source: Data Brief. 2017 May 17;13:536–44. doi: 10.1016/j.dib.2017.05.024 (PMC5491396; doi:10.1016/j.dib.2017.05.024)
Supplement: Supplementary file 5 — Table S1. Descriptions of the 18 selected NUMTs [file mmc5.pdf]

Supplementary material:

Fig. S4. Consensus trees of 18 selected NUMTs.

NUMT branches are colored red, inferred mitochondrial portions of the NUMT branches are green.

Please note that tree shapes provide only rough idea of the timing of the NUMT insertion. This is particular true in the case of NUMTs 21-22. Please note that estimating the time of insertion includes further steps beyond constructing the tree and estimation the mitochondrial portion of the NUMT branch.

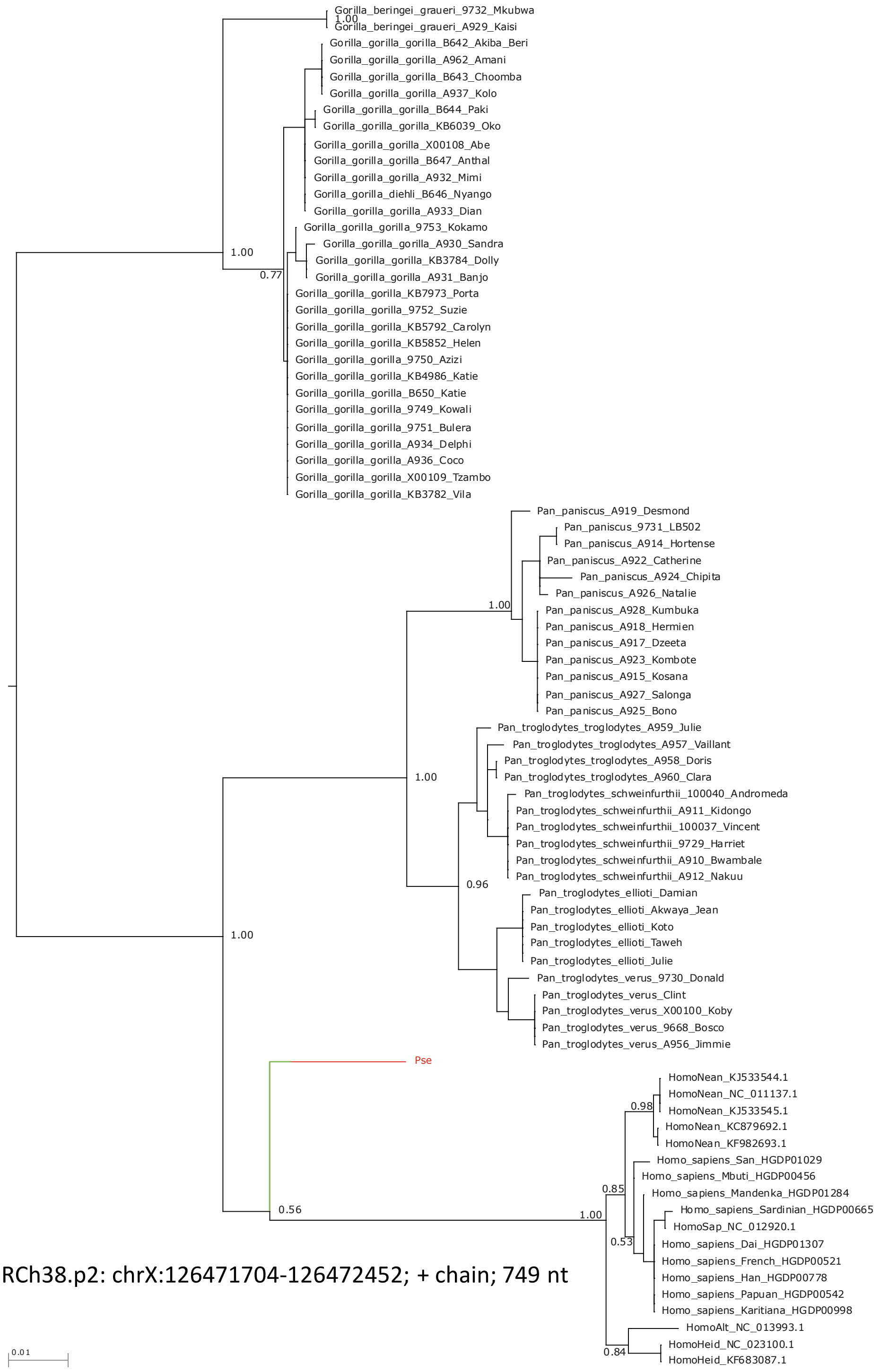

Pse_1: GRCh38.p2: chrX:126471704-126472452; + chain; 749 nt

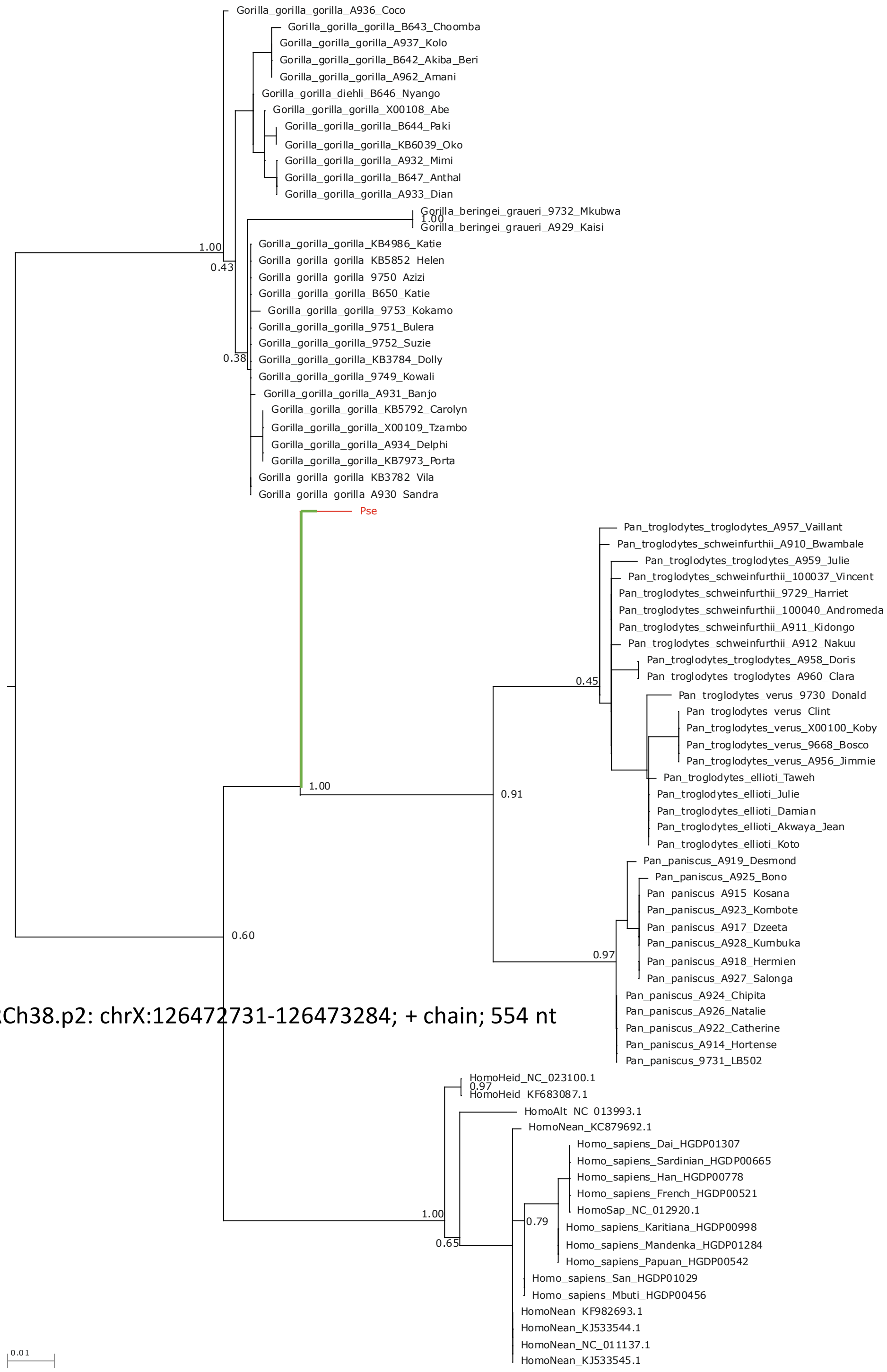

Pse_2: GRCh38.p2: chrX:126472731-126473284; + chain; 554 nt

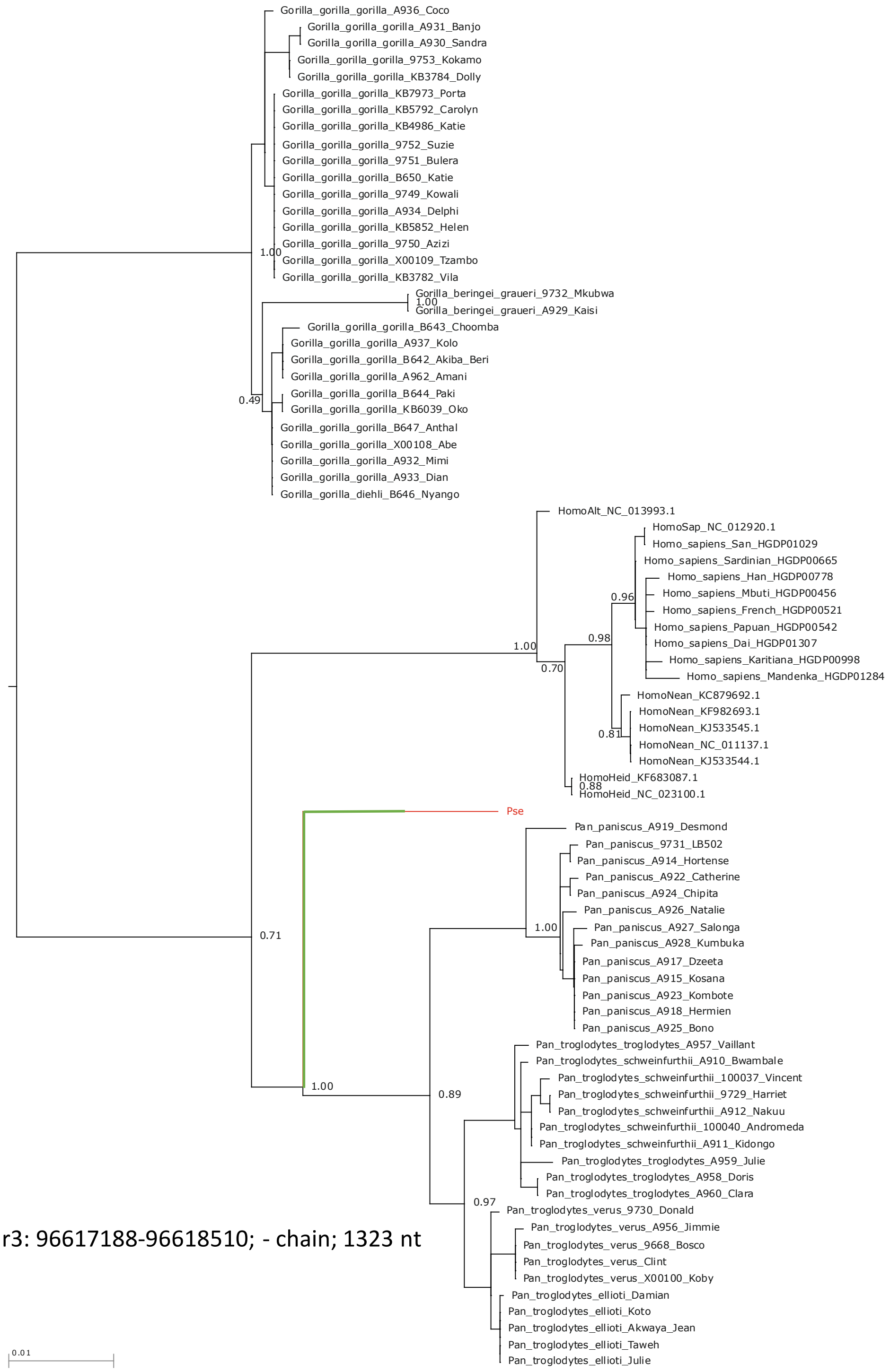

Pse_3: GRCh38.p2: chr3: 96617188-96618510; - chain; 1323 nt

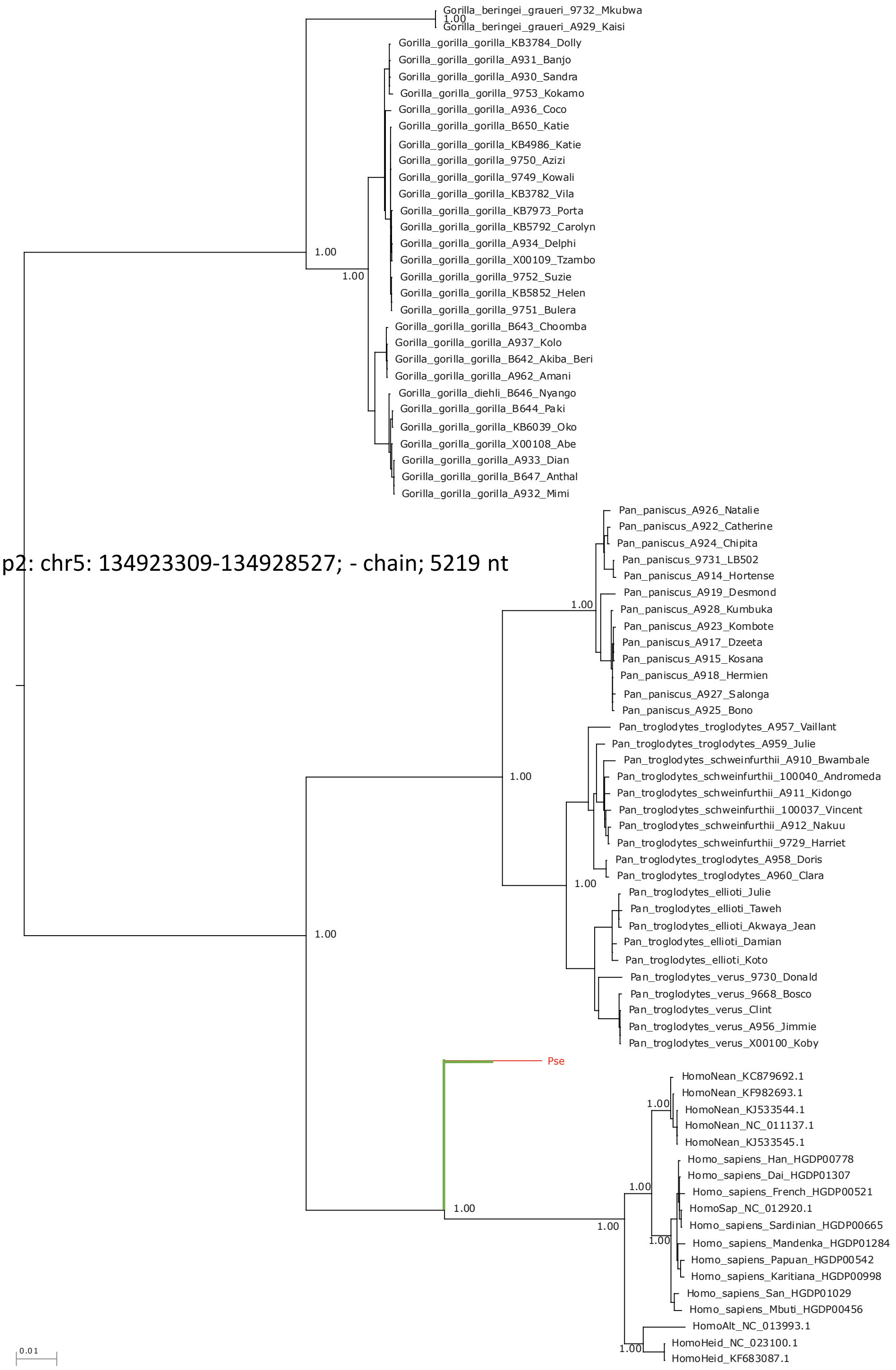
Pse_4: GRCh38.p2: chr5: 134923309-134928527; - chain; 5219 nt
Gorilla_beringei_graueri_9732_Mkubwa
Gorilla_beringei_graueri_A929_Kaisi
Gorilla_gorilla_gorilla_KB3784_Dolly
Gorilla_gorilla_gorilla_A931_Banjo
Gorilla_gorilla_gorilla_A930_Sandra
Gorilla_gorilla_gorilla_9753_Kokamo
Gorilla_gorilla_gorilla_A936_Coco
Gorilla_gorilla_gorilla_B650_Katie
Gorilla_gorilla_gorilla_KB4986_Katie
Gorilla_gorilla_gorilla_9750_Azizi
Gorilla_gorilla_gorilla_9749_Kowali
Gorilla_gorilla_gorilla_KB3782_Vila
Gorilla_gorilla_gorilla_KB7973_Porta
Gorilla_gorilla_gorilla_KB5792_Carolyn
Gorilla_gorilla_gorilla_A934_Delphi
Gorilla_gorilla_gorilla_X00109_Tzambo
Gorilla_gorilla_gorilla_9752_Suzie
Gorilla_gorilla_gorilla_KB5852_Helen
Gorilla_gorilla_gorilla_9751_Bulera
Gorilla_gorilla_gorilla_B643_Choomba
Gorilla_gorilla_gorilla_A937_Kolo
Gorilla_gorilla_gorilla_B642_Akiba_Beri
Gorilla_gorilla_gorilla_A962_Amani
Gorilla_gorilla_diehli_B646_Nyango
Gorilla_gorilla_gorilla_B644_Paki
Gorilla_gorilla_gorilla_KB6039_Oko
Gorilla_gorilla_gorilla_X00108_Abe
Gorilla_gorilla_gorilla_A933_Dian
Gorilla_gorilla_gorilla_B647_Anthal
Gorilla_gorilla_gorilla_A932_Mimi
Pan_paniscus_A926_Natalie
Pan_paniscus_A922_Catherine
Pan_paniscus_A924_Chipita
Pan_paniscus_9731_LB502
Pan_paniscus_A914_Hortense
Pan_paniscus_A919_Desmond
Pan_paniscus_A928_Kumbuka
Pan_paniscus_A923_Kombote
Pan_paniscus_A917_Dzeeta
Pan_paniscus_A915_Kosana
Pan_paniscus_A918_Hermien
Pan_paniscus_A927_Salonga
Pan_paniscus_A925_Bono
Pan_troglodytes_troglodytes_A957_Vaillant
Pan_troglodytes_troglodytes_A959_Julie
Pan_troglodytes_schweinfurthii_A910_Bwambale
Pan_troglodytes_schweinfurthii_100040_Andromeda
Pan_troglodytes_schweinfurthii_A911_Kidongo
Pan_troglodytes_schweinfurthii_100037_Vincent
Pan_troglodytes_schweinfurthii_A912_Nakuu
Pan_troglodytes_schweinfurthii_9729_Harriet
Pan_troglodytes_troglodytes_A958_Doris
Pan_troglodytes_troglodytes_A960_Clara
Pan_troglodytes_ellioti_Julie
Pan_troglodytes_ellioti_Taweh
Pan_troglodytes_ellioti_Akwaya_Jean
Pan_troglodytes_ellioti_Damian
Pan_troglodytes_ellioti_Koto
Pan_troglodytes_verus_9730_Donald
Pan_troglodytes_verus_9668_Bosco
Pan_troglodytes_verus_Clint
Pan_troglodytes_verus_A956_Jimmie
Pan_troglodytes_verus_X00100_Koby
Pse
HomoNean_KC879692.1
HomoNean_KF982693.1
HomoNean_KJ533544.1
HomoNean_NC_011137.1
HomoNean_KJ533545.1
Homo_sapiens_Han_HGDP00778
Homo_sapiens_Dai_HGDP01307
Homo_sapiens_French_HGDP00521
HomoSap_NC_012920.1
Homo_sapiens_Sardinian_HGDP00665
Homo_sapiens_Mandenka_HGDP01284
Homo_sapiens_Papuan_HGDP00542
Homo_sapiens_Karitiana_HGDP00998
Homo_sapiens_San_HGDP01029
Homo_sapiens_Mbuti_HGDP00456
HomoAlt_NC_013993.1
HomoHeid_NC_023100.1
HomoHeid_KF683087.1
1.00
0.01

Pse_5: GRCh38.p2: chr14: 32484098 -32485118; - chain; 1021 nt

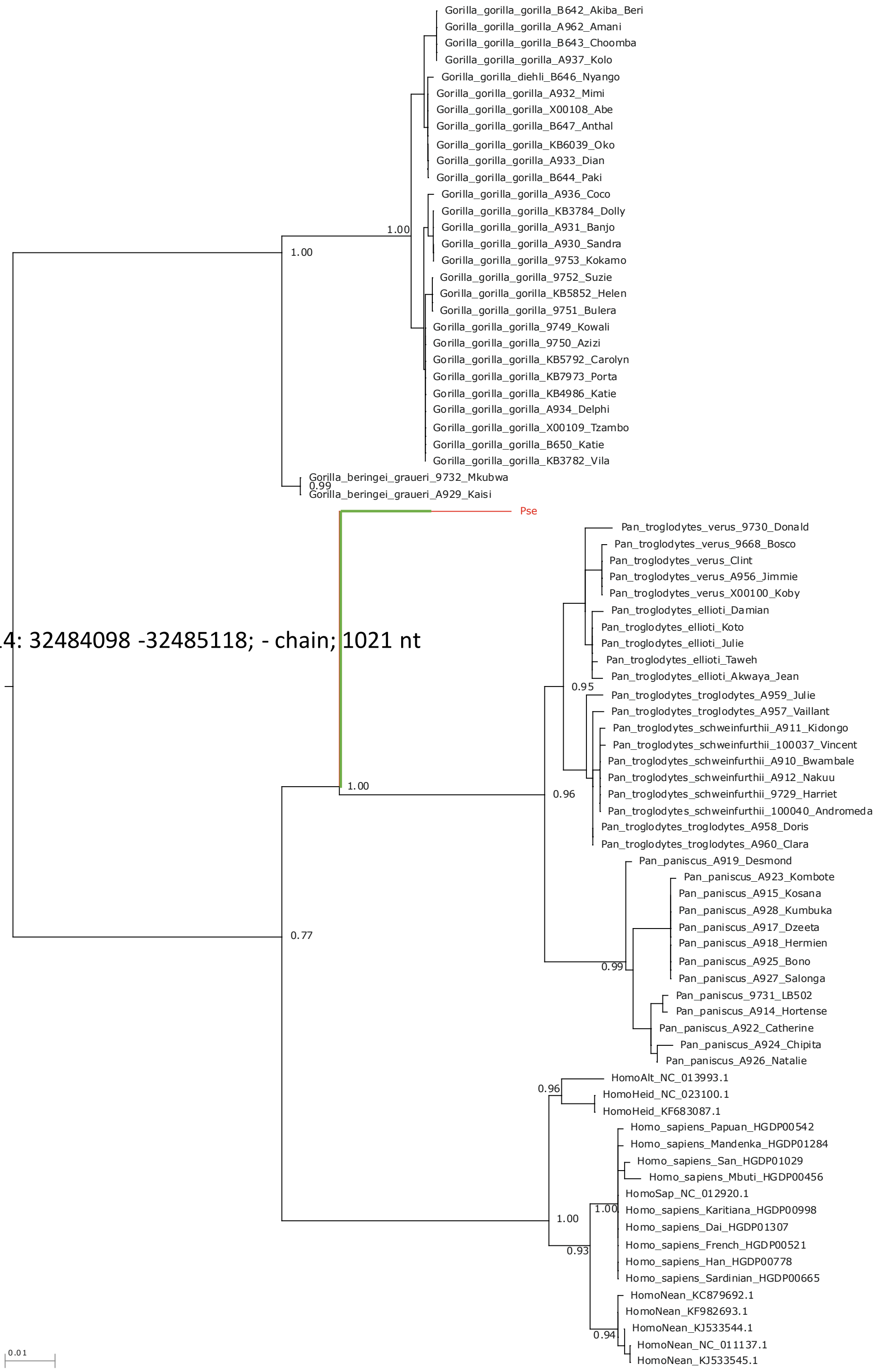

Pse_6: GRCh38.p2: chr17: 53105733-53106385; + chain; 653 nt

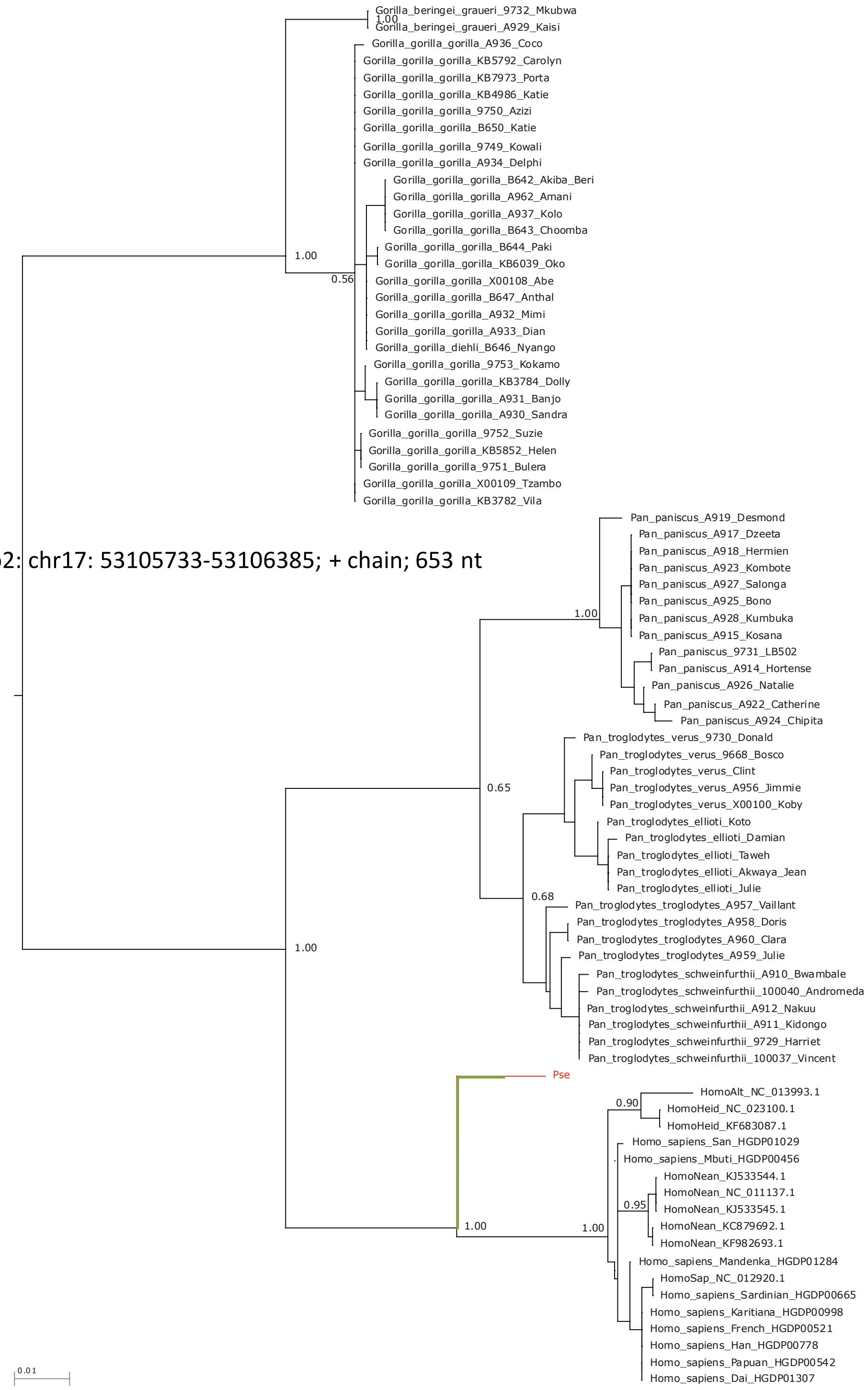

Pse_7: GRCh38.p2: chr5:80650022-80652135; - chain; 2114 nt

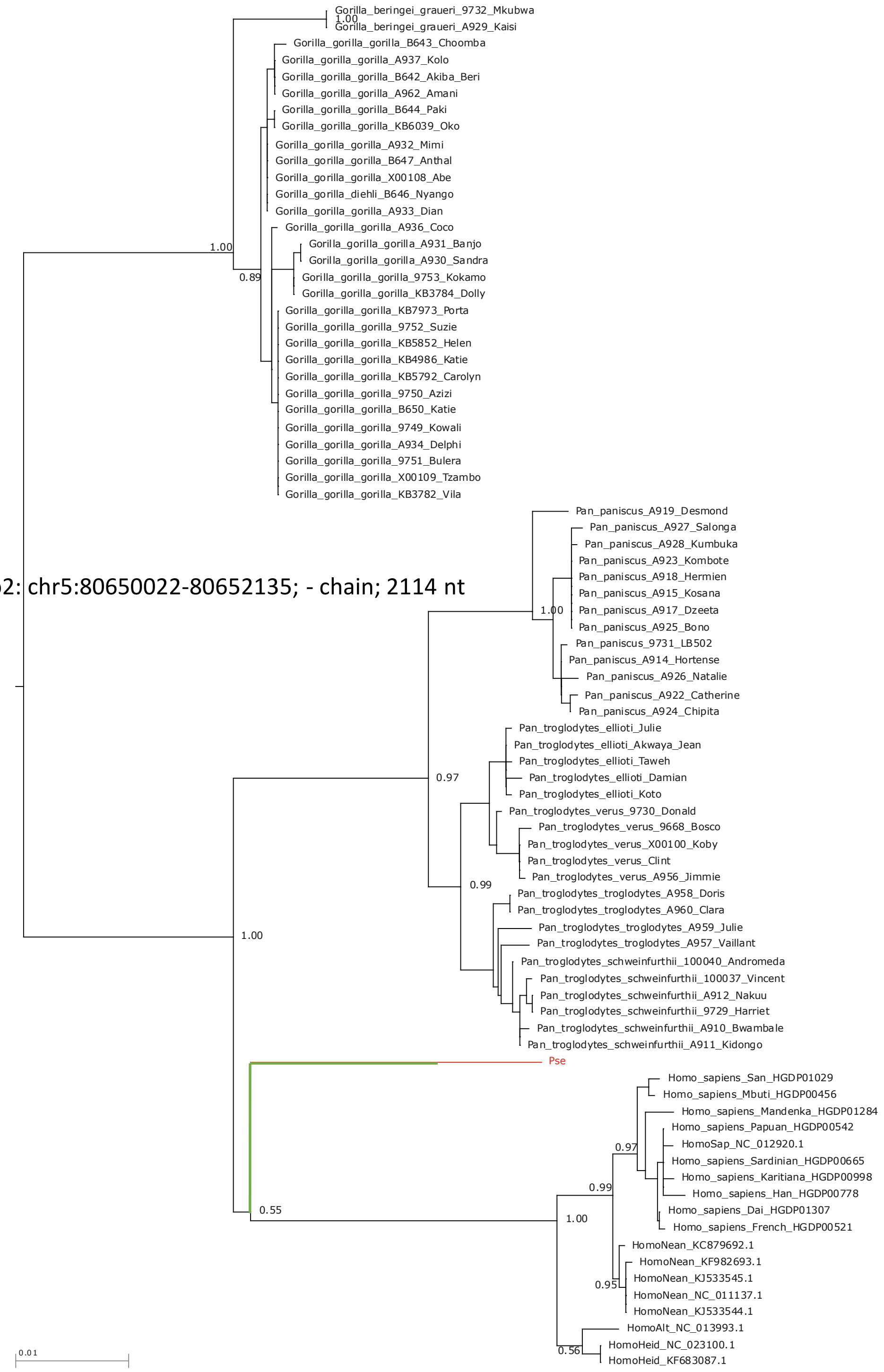

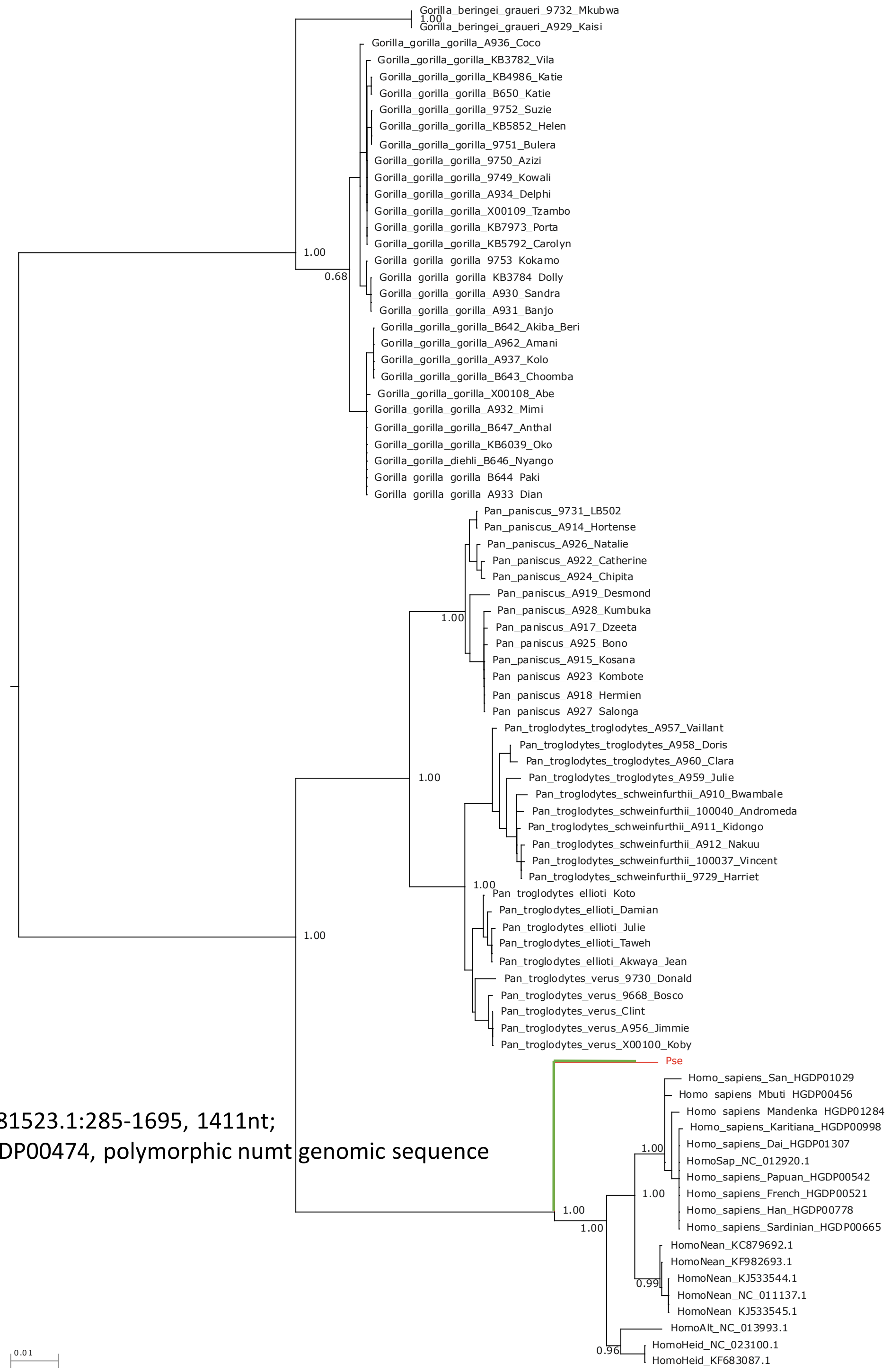

Pse_8: GenBank ID: KM281523.1:285-1695, 1411nt;
Homo sapiens isolate HGDP00474, polymorphic numt genomic sequence

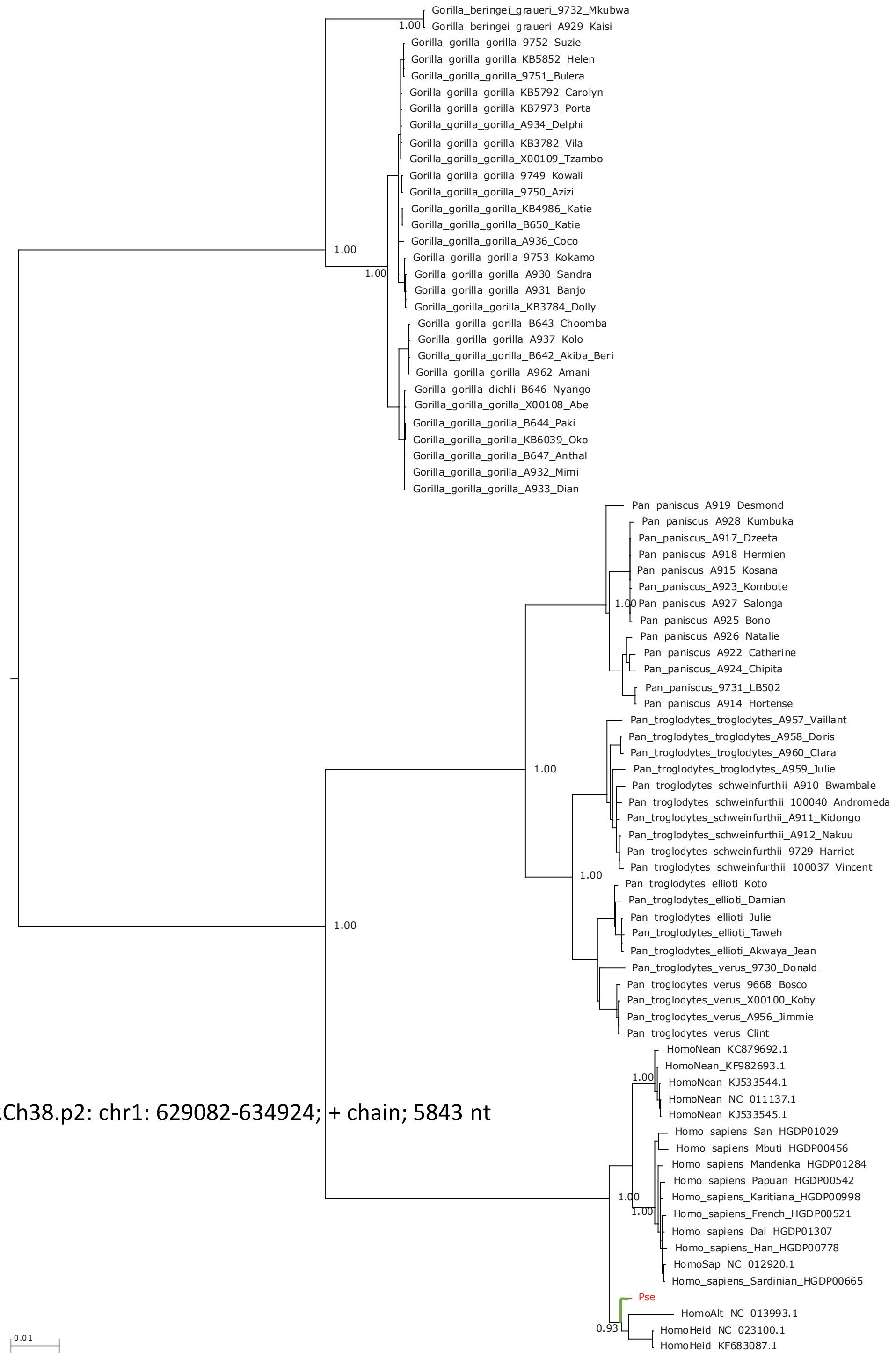

Pse_9: GRCh38.p2: chr1: 629082-634924; + chain; 5843 nt

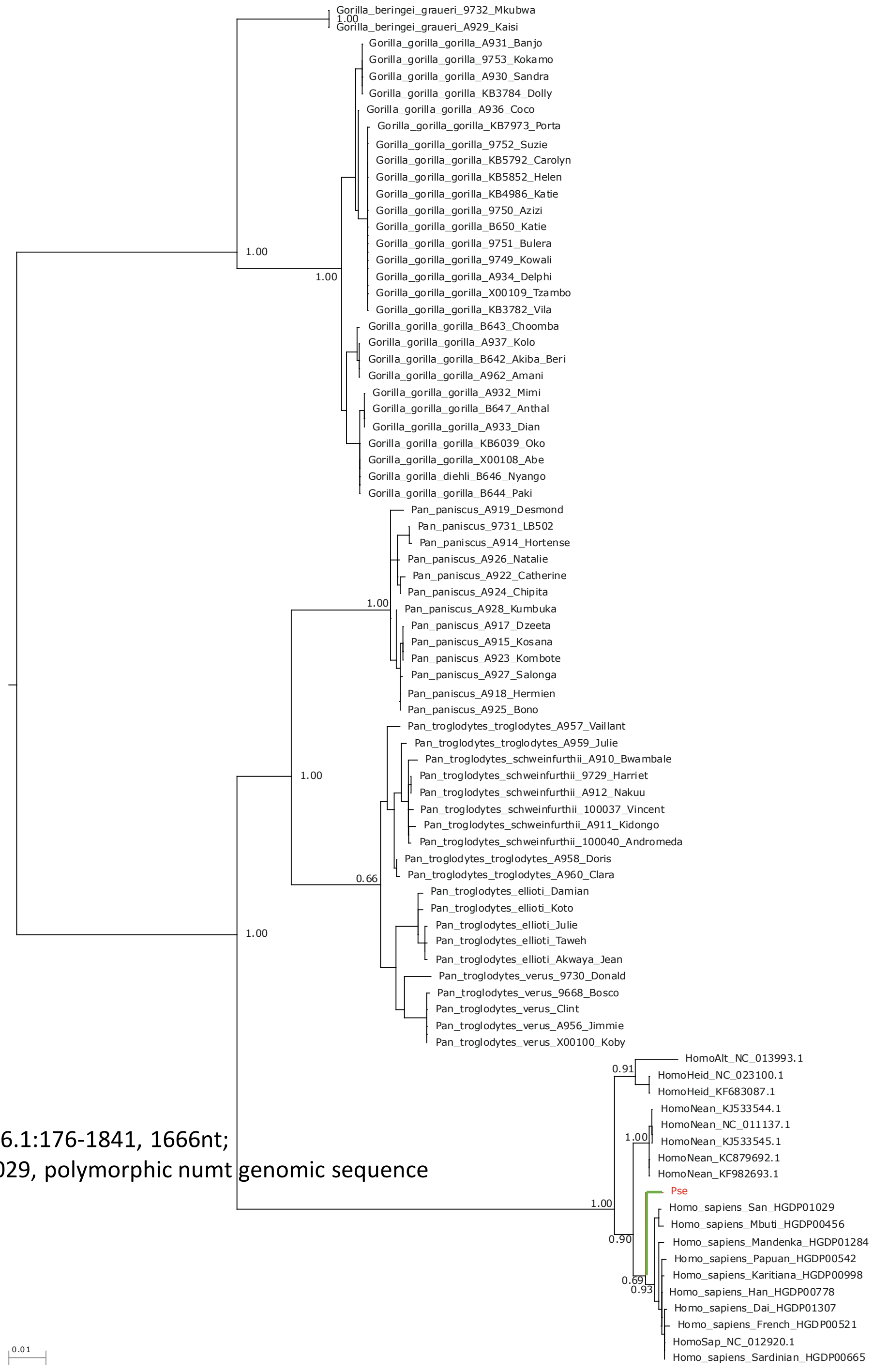

Pse_10: GenBank ID: KM281526.1:176-1841, 1666nt;
Homo sapiens isolate HGDP01029, polymorphic numt genomic sequence

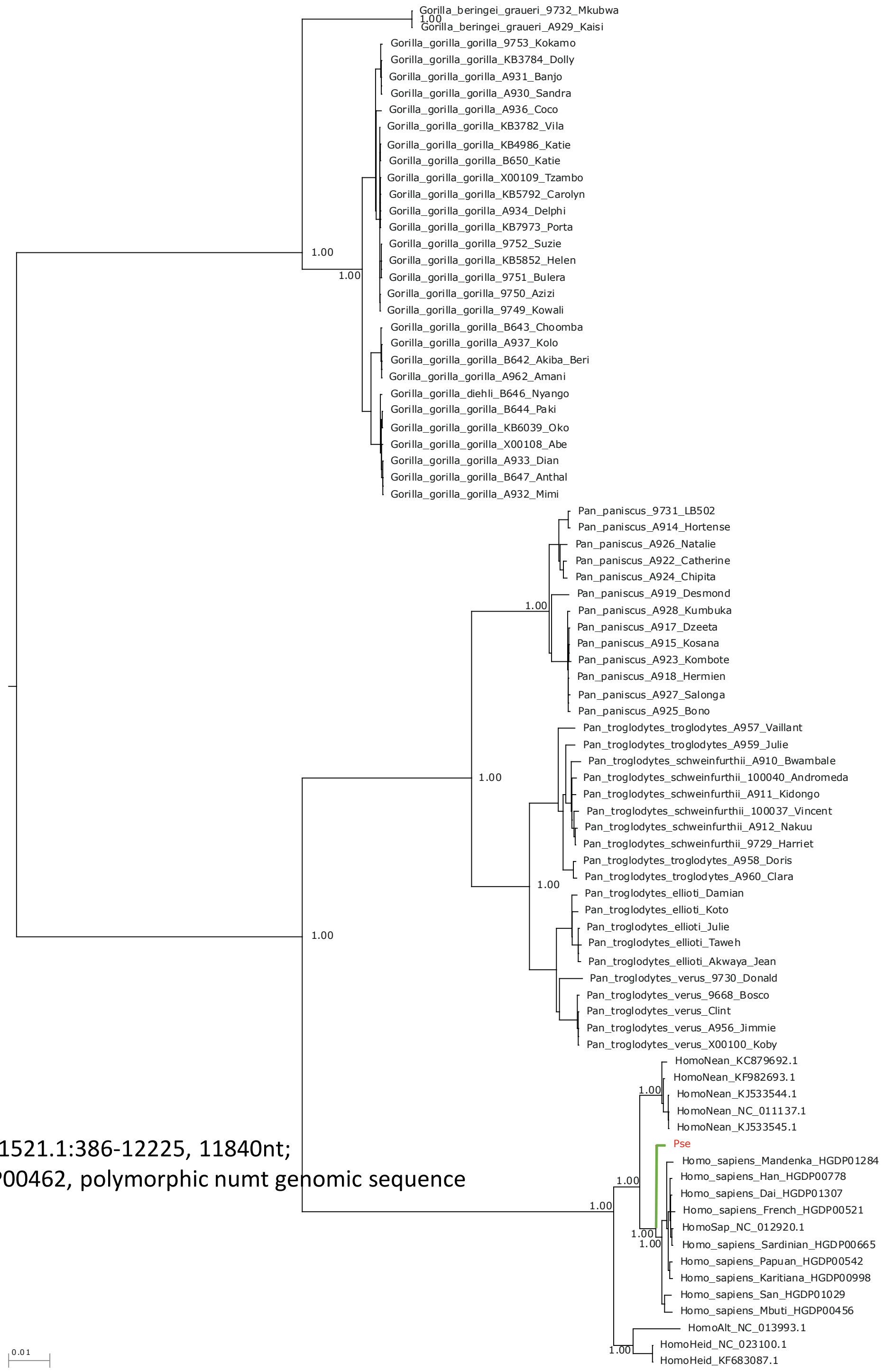

Pse_11: GenBank ID: KM281521.1:386-12225, 11840nt;
Homo sapiens isolate HGDP00462, polymorphic numt genomic sequence

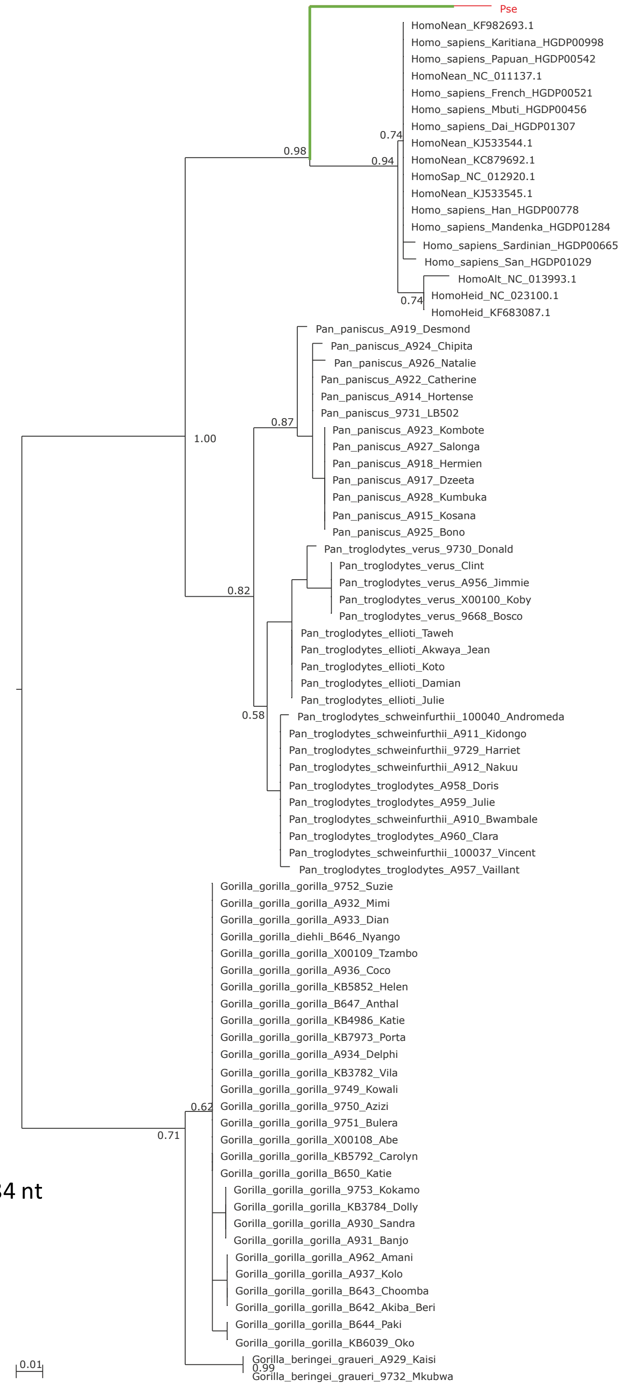

Pse_14: GRCh38.p2: chr2:49229628-49229911; - chain; 284 nt

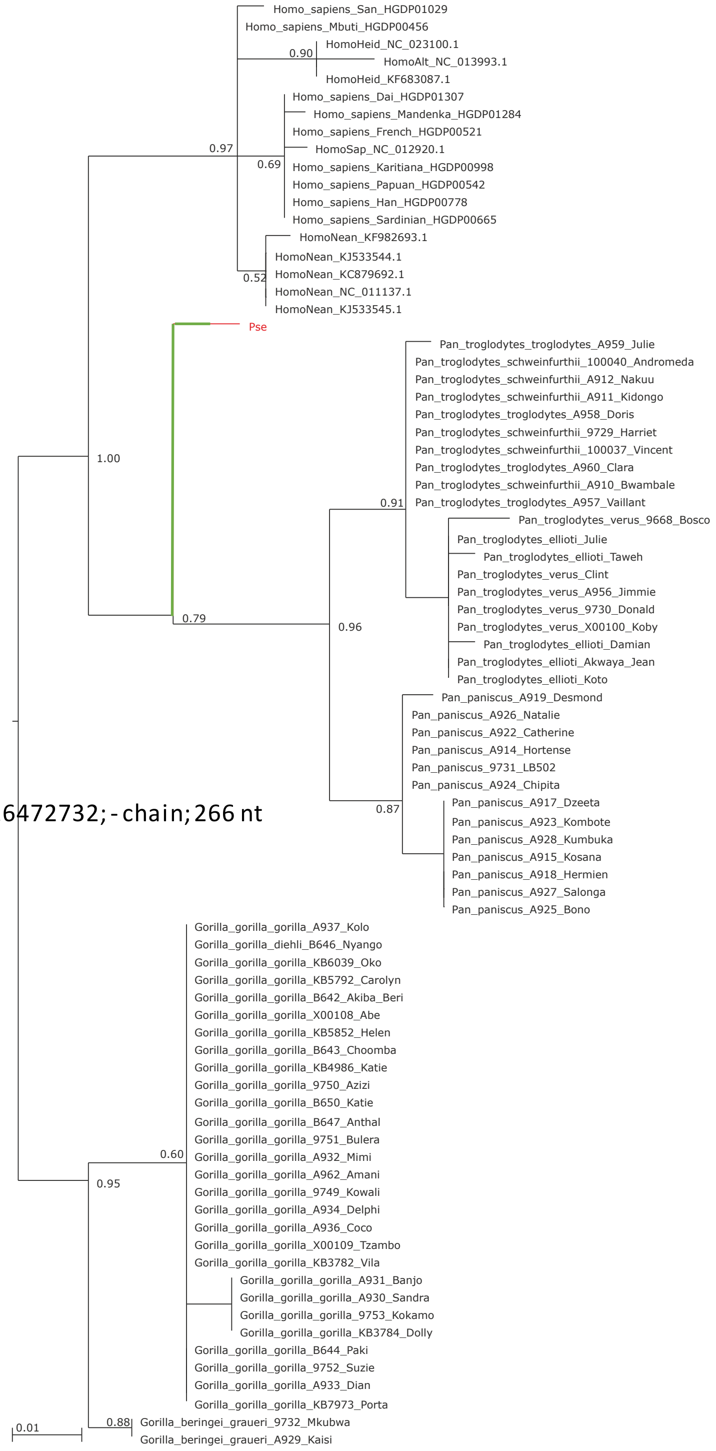

Pse_15: GRCh38.p2: chrX:126472467-126472732; - chain; 266 nt

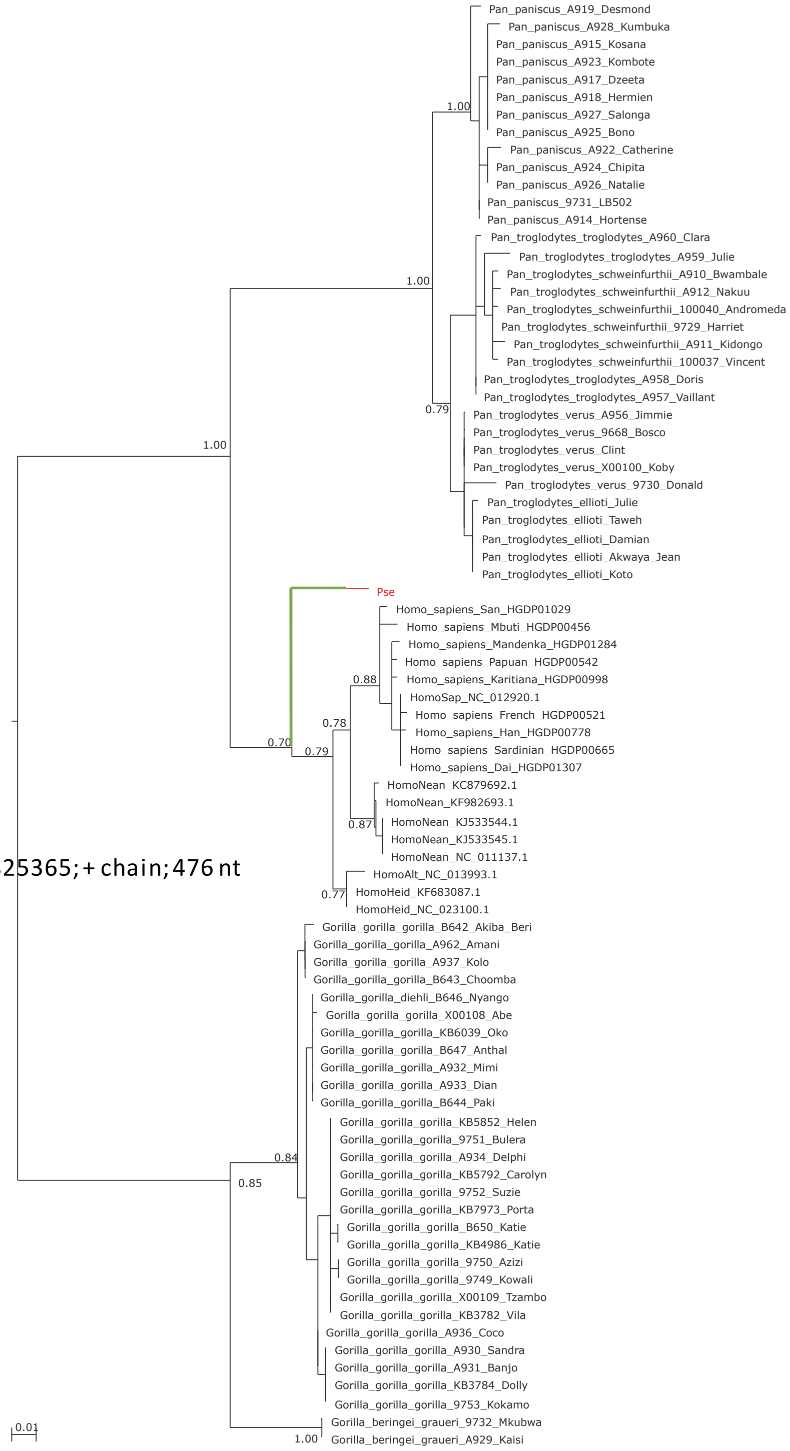

Pse_16: GRCh38.p2: chr2:87824890-87825365; + chain; 476 nt

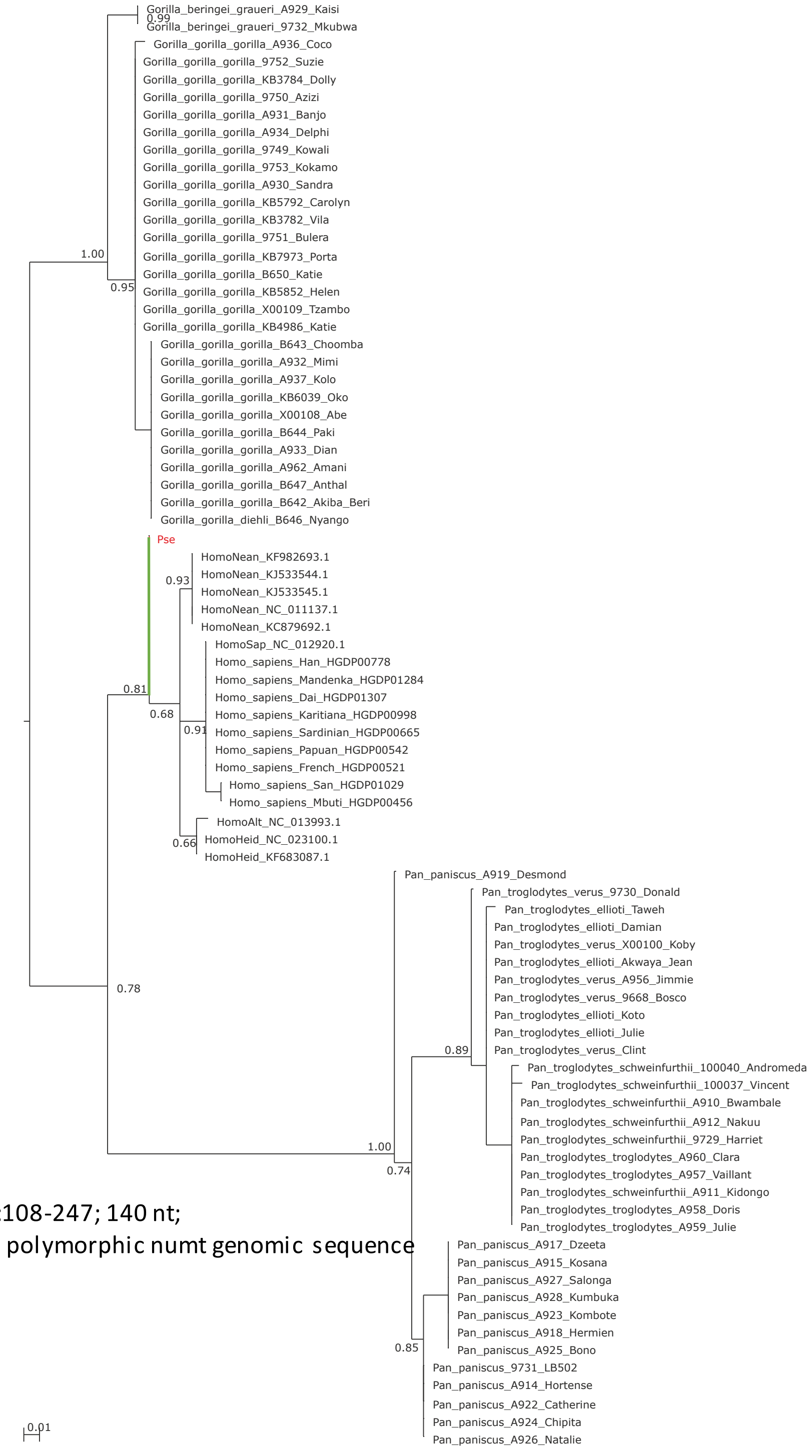

Pse_17: GenBank ID: KM281531.1:108-247; 140 nt;
Homo sapiens isolate HGDP00992, polymorphic numt genomic sequence

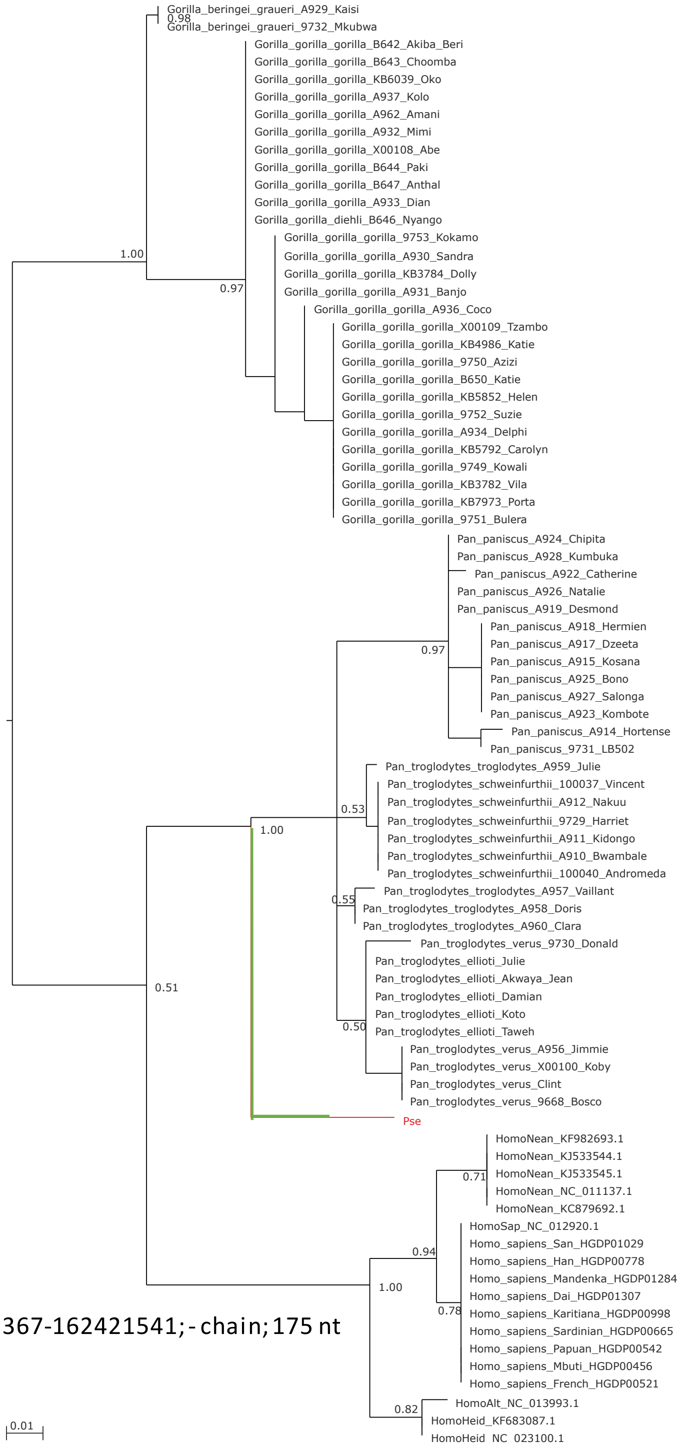

Pse_19: GRCh38.p2: chr4:162421367-162421541; - chain; 175 nt

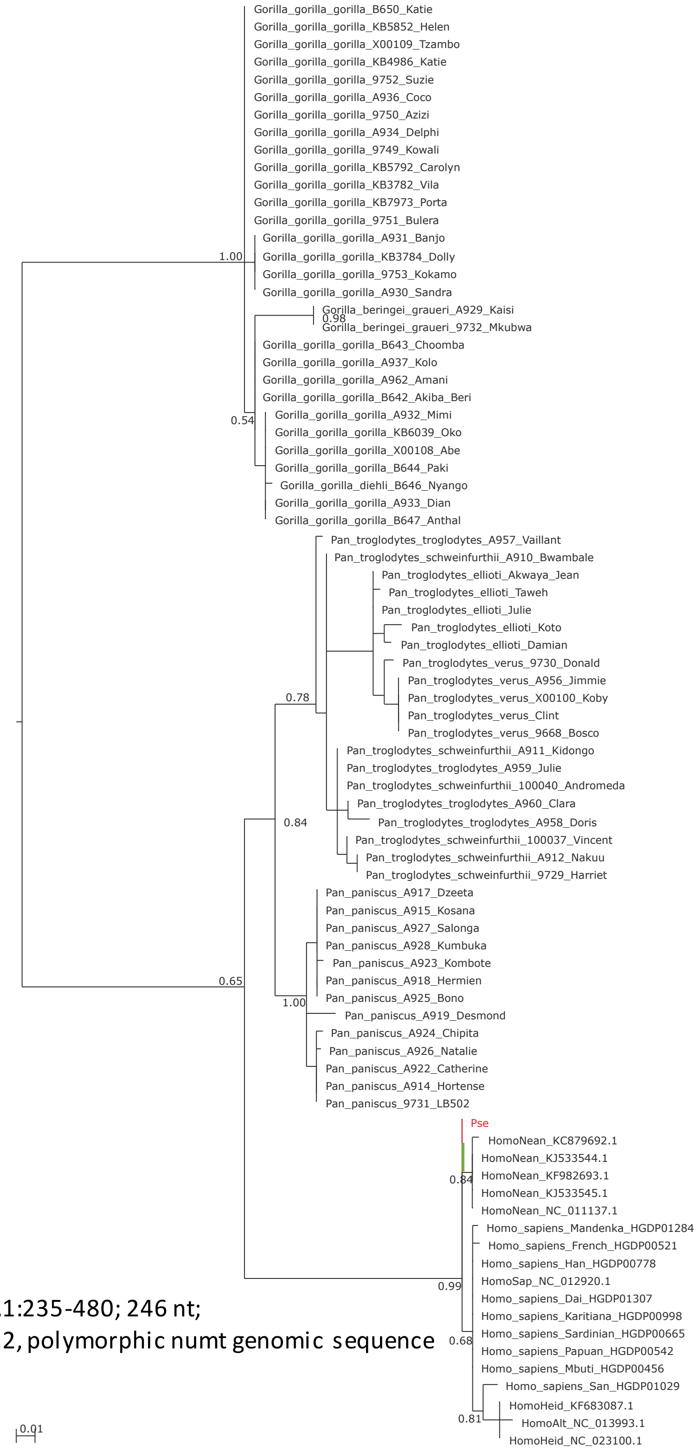

Pse_21: GenBank ID: KM281513.1:235-480; 246 nt;
Homo sapiens isolate HGDP00222, polymorphic numt genomic sequence

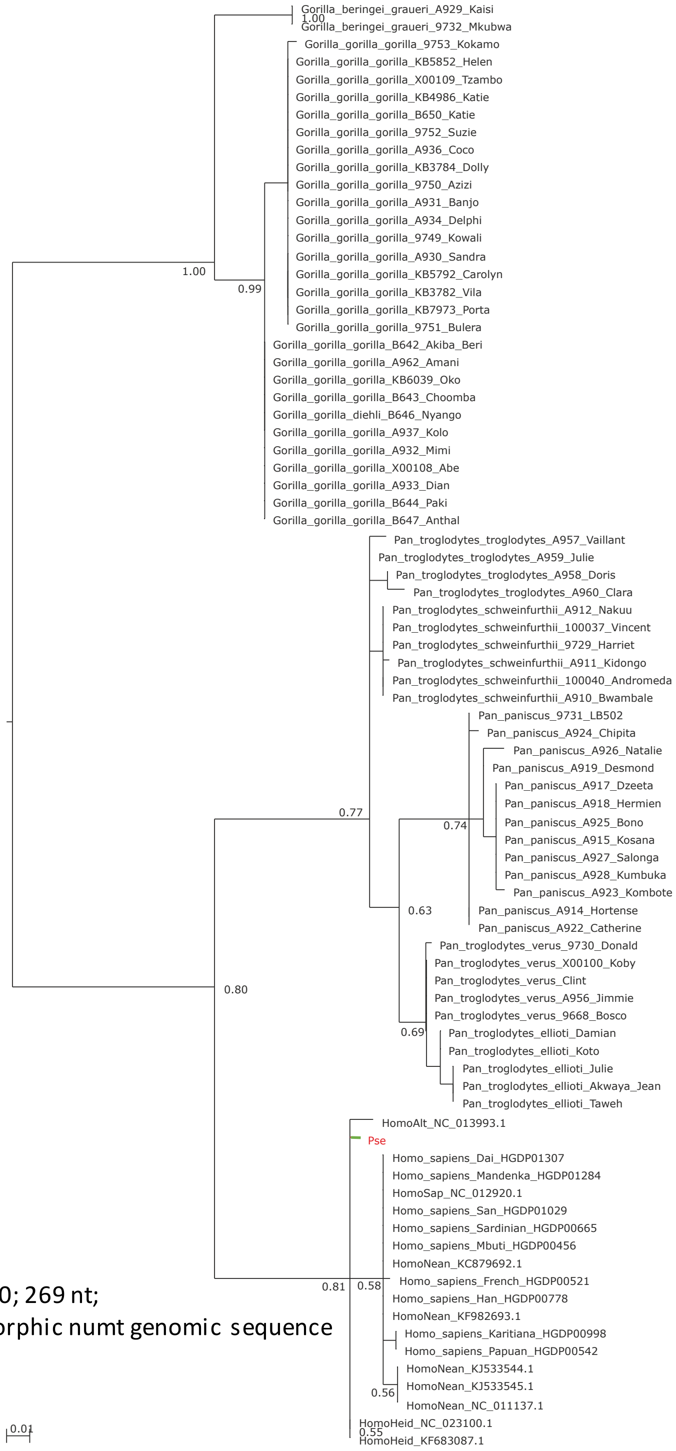

Pse_22: GenBank ID: KM281530.1:172-440; 269 nt;
Homo sapiens isolate HGDP01032, polymorphic numt genomic sequence

Sequences of the 18 selected NUMTS.

>Pse_1
CCACCTTCTTCGACCCAGCCGGGGGAGGGGACCCTATTCTATACCAACACCTATTCTGATTTTTTGGCCACCCCG
AAGTTTATATTCTTATCCTGCCAGGCTTCGGAATAATTTCCCATATTGTAACTTACTACTCCGGAAAAAAAGAA
CCATTCGGATATATAGGTATGGTCTGAGCTATAATATCAATTGGTTTCTTAGGGTTTATTGTGTGAGCACACC
ATATATTTACAGTAGGAATGGACGTAGACACACGAGCCTATTTCACCTCCGCTACCATAATCATCGCTATTCCC
ACCAGCGTCAAAGTATTTAGCTGACTCGCTACACTCTACGGAAGCAATATGAAGTGATCTGCTGCAGTACTCTG
AGCCCTAGGGTTCATTTTTCTCTTCACTGTAGGTGGCCTAACCGGCATTGTACTAGCAAACTCATCATTAGACA
TCGTACTACACGACACATACTACGTCGTAGCTCACTTCCATTACGTCCTATCAATAGGAGCTGTATTCGCCATC
ACAGGAGGTTTCATTCACTGATTTCCCCTGTTCTCAGGCTATACCCTAGACCAAACCTACGCCAAATCCATTTT
GCTATCATATTCATTGGCGTAAACCTAACCTTCTTCCCACAACACTTTCTTGGCCTATCTGGAATACCCCGACG
TTACTCGGACTACCCCGATGCATACACCACATGAAATATCCTATCATCTGTAGGCTCATTCATTTCCCTAACAG
CAGTAATAT
>Pse_2
TCATAACCCTCAACACCCACTCCCTCTTAGCCAATATTGTACCTATCACCATACTAGTCTTTGCTGCCTGCAAGG
CAGCAGTAGGCCTAGCCCTACTAGTCTCAATCTCTAACACATATGGCCTAGACTACGTACATAACCTAAGCCTA
CTCCAATGCTAAAACTAATCATCCCAACAATCATATTACTACCACTAACATGATTCTCCAAAAAACATATAATT
TGAATCAACACAACCACTCACAGCCTAATTATTAGCACCATCCCCCTACTATTTTTTAACCAAATCAACAACAA
CCTATTTAGCTGCTCCCTATCCTTCTCCTCCGACCCCCTAACGACCCCCCTCCTAATACTAACTACCTGACTTCT
ACCCCTCACAATCATGGCAAGCCAGCGCCACCTATCCAACGAACCACTATCACGAAAAAAACTCTACCTCTCTAT
GCTAATCTCCCTCCAAATCTCCTTAATTATAACATTCACAGCCACGGAGCTAATCATATTTTATATCTTCTTCG
AAACCACACTTATCCCCACCCTAGCTATCATCA
>Pse_3
CTTATGAAATTTAAGGGTCGAAGGTGGATTTAGCAGTAAACTAAGTGTAGAGTGCTTAGTTGAACAGGGCCCT
GAAGCGCATACACACCGCCCGTCACCCTCCTCAAGTATACTTCAAAGGATATTTAACTAAAACCCCTACGCATT
TATATAGAGGAAACAAGTCGTAACATGGTAAGTGTAGTGGAAAGTGCACTTGGACGAACCAGAGTGTAGCTTA
ACATAAAGCACCCAACTTACACTTAGGAGATTTCAACTCAACTTGACCACTCTGAGCCAAACCTAGCCCTAAAC
CCGTTCCACCTTACTATCAAATAACCTTAACCAAACCATTTACCCAAATAAAGTATAGGCGATAGAAATTGTAA
ACCGGCGCAATAGATATAGTACCGCAAGGGAAAGATGAAAAATTATAACCAAGCATAATACAGCAAGGACTAA
CCCCTGTACCTTTTGCATAATGAATTAACTAGAAATAACTTTGCAAAGAGAACCAAAGCTAAGGCCCCCGAAAC
CAGACGAGCTACCTAAGAACAGCTAAAAGAGCACACCCGTCTATGTAGCAAAATAGTGGGAAGATTTATAGGT
AGAGGCGACAAACCTATCGAGCCTGGTGATAGCTGGTTGTCCAAGATAGAATCTTAGTTCAACTTTAAATTTAC
CTACAGAACCTTCTAAATCCCCTTGTAAATTTAACTGTTAGTCCAAAGAGGAACAGCTCTTTGGACACTAGGAA
AAAACCTTGTAAAGAGAGTAAAAAATTTAATACCCATAGTAGGCCTAAAAGCAGCCACCAATTAAGAAAGCGT
TCAAGCTCAACACCCATCGTCTAAAAAATCCCAAACATACAACTGAGCTCCTTACACTCAATTGGACCAATCTA
TTACCTTATAGAAGAACTAATGTTAGTATAAGTAACATGAAAACATTCTCCTCCGCATAAGCCTACATCAGACC
AAAATATTAAACTGACAATTAACAGCCCAATATCTACAATCAACCAACAAGCCATTATTACCCTCACTGTTAAC
CCAACACAGGCATGCCCACAAGGAAAGGTTAAAAAAAGTAAAAGGAACTCGGCAAATCTTACCCCGCCTGTTTA
CCAAAAACATCACCTCTAGCATTATCAGTATTAGAGGCACCGCCTGCCCGGTGACATATGTTTAACGGCCGCGG

TACCCTAACCGTGCAAAGGTAGCATAATCACTTGTTCCTTAAATAGGGACTTGTATGAATGGCTCCACGAGGGT
TCAGCTGTCTCTTACTTTCAACCAGTGAAATTGACCTACCCGTGAAGAGGCGGGCATAACATAGCAAGAC
>Pse_4
CTCCTTTTACCCTTGCCATGAGCCCTACAAACAACTAACTTACCACTAATAGTCACATCGTCCCTCTTATTAATC
ATCATCCTAGCCCTAAGTCTGGCCTACGAATGATTATAAAAAGGGTTAGACTGAGCCGAATTGGTATATAGTTC
AAACAAAACGAATGATTTCGACTCATTAAATTATGATAATCATATTTACCAAATGCCTCTCATTTACATAAATA
TTATACTAGAATTTACCATCTCACTTCTAGGAATACTAGTATATCGCTCACACCTGATATCCTCCCTACTATGC
CTAGAAGGAATAATACTATCACTATTCATTATAGCCACTCTCATAACCCTCAACACCCACTCCCTCTTAGCCAA
TATTGTACCTATCGCCATACTAGTCTTTGCTGCCTGCAAGGCAGCAGTAGGCCTAGCCCTACTAGTCTCAATCT
CCAACACGTATGGCCTAGACTACGTACATAACCTAAGCCTACTCCAATGCTAAAACTAATTGTCCCAACAATTA
TATTACTACCACTGACATGACTCTCCAAAAAACACATAATTTGAATCAACACAACCACTCACAGCCTAATTATT
AGCACCATCCCCCTACTATTTTTCAACCAAATCAACAACAACCTATTTAGCTGCTCCTTATCCTTCTCCTCCGAC
CCCCTAACGACCCCCCTCCTAATACTAACTACCTGACTCCTGCCCCTCACAATCATGGCAAGCCAACGCCACTTA
TCCAATGAACCACTATCACGAAAAAAACTCTACCTCTCTATACTAATCTCCCTACAAATCTCCTTAATTATGAC
ATTCACAGCCATAGAACTAATCATATTTTATATCTTCTTCGAAACCACACTTATCCCCACCCTGGCTATCATCA
CCCGATGAGGCAACCAACCAGAACGCCTGAACGCAGGTACATACTTCCTATTCTACACCCTAGTAGGCTCCCTCC
CCCTACTCATCGCACTAATCTACACCCACAACACCCTAGGCTCACTGAATATTCTATTACTCACTCTTACTGCCC
AAGAACTATCAAACTCCTGAGCCAATAACTTAATATGGCTAGCGTACACAATAGCTTTTATAGTAAAAATACCT
CTTTACGGTCTCCACCTATGGCTCCCTAAAGCCCATGTCGAAGCCCCCATCGCTGGGTCAATAGTACTTGCTGCA
GTACTCTTAAAATTAGGCGGCTATGGTATAATACGCCTCACACTCATTCTCAACCCCCTGACAAAACATATAGC
CTACCCCTTCCTTGTACTATCCCTGTGAGGCATAATTATAACAAGCTCCATCTGCCTGCGACAAACAGACCTAA
AATCGCTCATTGCATACTCTTCAATCAGCCACATAGCCCTCGTAGTAACAGCCATCCTCATCCAAACCCCCTGAA
GCTTCACCGGCGCAGTCATTCTCATAGTCGCCCACGGACTTACATCCTCATTACTATTCTGCCTAGCAAACTCAA
ACTACGAACGTACCCACAGTCGCATCATAATTCTCTCTCAAGGACTTCAAACCCTACTCCCACTAATAGCCTTTT
GATGACTTCTAGCAAGCCTCACTAATCTCGCCTTACCCCCCACTATTAACCTACTAGGAGAACTCTCTGTGCTA
GTAACCACATTCTCCTGATCAAATATCACTCTCCTACTTACAGGACTCAACATACTAATCACAGCCCTATACTC
CCTCTACATATTTACCACAACACAATGAGGCTCGCTCAGCCACCACATTAACAACATAAAACCCTCATTCACAC
GAGAAAACACTCTCATGTTCATACACCTATCCCCTATCCTCCTCCTATCCCTCAACCCCGATATCATTACCGGGT
TTTCCTCCTGTAAATATAGTTTAACCAAAACATCAGATTGTGAATCTGACAACAGAGGCTCACGACCCCTTATT
TACCGAGAAAGCGCATAAGAACTGCTAACTCATATCCCCATGTCTAACAACATGGCTTTCTCAACTTTTAAAGG
ATAACAGCCATCCGTTGGTCTTAGGCCCCAAAAATTTTGGTGCAACTCCAAATAAAAGTAATAACCATGTACGC
TACTATACCCACCCTAGCCCTAACTTCCTTAATTCCCCCTATCCTTACCACCCTCATTAACCCTAATAAAAAAA
CTCATATCCCCATTAAGTAAAATCCATTATCGCATCCACCCTTGTTATTAGTCTCTTCCCCACAACAATATTCA
TATACCTAGACCAAGAAGTCATTATCTCAAACTGACACTGGGCAACAACCCAAACAACCCAACTCTCCCTAAGC
TTCAAACTAGATTATTTCTCCATAATATTTATCCCTGTAGCACTGTTCGTTACGTGATCCATCATAGAATTCTC
ACTATGATATATAAGCTCAGACCCAAACATTAATCAATTCTTCAAATACCTACTCATCTTCCTAATCACCATAC
TAATCCTAGTCACTGCTAACAACCTATTCCAGCTATTCATCGGCTGAGAAGGCGTAGGAATTATATCCTTTTTA
CTCATTAGTTGATGATACGCCCGAGCAGATGCCAACACAGCAGCTATTCAAGCAATCCTATACAACCGTATCGG
CGACATCGGTTTCATCCTTGCCCTAGCATGATTTCTCCTACACTCCAACTCATGAGACCCACAACAAATAACCCT
CCTAAGTGCTAATCCAAGCCTCACCCCACTACTAGGCTTCCTCCTAGCAGCAGCAGGCAAATCAGCTCAATTAG
GCCTTCACCCCTGACTCCCCTCAGCCATAGAAGGCCCCACCCCTGTCTCAGCCCTACTCCACTCAAGCACTATAG
TTGTAGCAGGAGTCTTCCTACTCATCCGCTTCCACCCCCTAGCAGAGAATAACCCACTAATCCAAACTCTCACAC
TATGCCTAGGCGCTATCACCACTCTGTTCGCAGCAGTCTGCGCCCTTACACAAAATGACATCAAAAAAATCGTG

GCCTTCTCCACTTCAAGCCAACTAGGACTTATAATAGTCACAATCGGCATCAACCAACCACACCTAGCATTCCT
GCACATCTGTACCCACGCCTTCTTCAAAGCCATACTATTTATATGCTCCGGATCCATCATCCACAACCTCAACAA
TGAACAAGATATTCGAAAAATAGGAGGACTACTCAAAACCATACCTCTTACTTCAACCTCCCTCACCATTGGCA
ACCTAGCATTGGCAGGAATACCCTTCCTCACAGGTTTCTACTCCAAAGACCACATCATCGAAACCGCAAACATA
TCATACACAAACGCCTGAGCCCTGTCTATTACTCTCATCGCCACCTCCCTGACAAGCGCCTATAGCACTCGAATA
ATTCTTCTCACCCTAACAGGTCAGCCTCGCTTCCCAGCCCTCACTAACATTAACGAAAACAACCCCACCCTACTA
AACCCCATTAAACGCCTAACAATCGGAAGCCTATTTGCAGGGTTTCTCATCACCAACAGCATTCTCCCCACATCC
ACCCCCCAAATGACAATCCCACTTTACTTAAAACTCACAGCCCTAGGCGTCACTTCCCTAGGACTTCTAACAGCC
CTAGATCTCAACTACCTAACCAACAAACTCAAAATAAAATCCCCACTGTATACATTTTACTTCTCTAATATACT
CGGATTTTACCCTAGTATCATACACCGCACAGTCCCTTATCTAGGCCTTCTTACAAGCCAAAACCTACCCCTACT
CCTCCTAGACCTGGCCTGACTAGAAAAGCTACTACCTAAAACAATCTCACAACACCAAATCTCCGCCTCCATCAT
CACCTCAACCCAAAAAGGCATAATCAAACTTTATTTCCTCTCTTTCTTCTTCCCACTCATCCTAACCCTACTCCT
AATCACATAATCTATTCCCCCGAGCAATCTCTAGTACAATATACACACCAACAAACAATGTTCAACCAGTAACT
ACTACTAATCAACGCCCATAATCATACAAAGCCCCCGCACCAATAGGATCCTCCCGAATCAGCCCTGACCCTTCT
CCTTCATAAATTATTCAACTTCCCACACTATTAAAATTTACCACAACCACCACCCCATCATACTCTTTCACCCAT
AACACTAATCCTACTTCCATCACCAACCCCACTAAAACACTTACCAAGACCTCAACCCCTGACCCCCATGCCTCA
GGATACTCCTCAATAGCCACCGCTGTAGTATATCCAAAAACAACCATCATTCCCCCCAAATAAATTAAAAAAAC
CATTAAACCCATGTAACCTCCCCCGTAATTCAGAATAATAACACACCCAATCACACCACTAACAATCAATACTA
AACCCCCATAAATAGGAGAAGGCTTAGAAGAAAACCCCACAAACCCCATTACTAAACCCACACTCAACAGAAAT
AAAGCATACATCATTATTCTTGCACGGGCTACAACCACGACCAATGATATGAAAAACCATCATTGTATTTCAAC
TACAAGAACACTAATGACCCCAATACGCAAAATTAACCCCTTGATAAAATTAATTAACCGCTCATTTATCGACC
TCCCCACCCCATCCAATATCTCCGCATGATGAAACTTCGGCTCACTTCTTGGTGCCTGCCTAATCCTCCAAATCA
CCACAGGACTATTCCTAGCCATACACTACTCACCAGACGCTTCAACCGCCTTCTCATCGATCGCCCATATCACCC
GAGACGTAAATTATGGCTGAATCATCCGCTACCTTCACGCCAATGGCGCCTCAATATTCTTCATCTGCCTCTTC
CTACACATCGGCCGAGGCCTATATTACGGCTCATTTCTTTACCTAGAAACCTGAAACATTGGCATCATCCTCCT
ACTTACAACTATAGCAACAGCCTTCATGGGCTATGTCCTCCCATGAGGCCAAATATCCTTCTGAGGGGCCACAG
TAATTACAAACCTGCTATCCGCCGTCCCATACATCGGAACAGATCTGGTCCAATGAATCTGAGGAGGCTACTCA
GTAGATAGTCCCACCCTCACACGGTTCTTTACCTTTCACTTTATCTTGCCCTTCATTATCACAGCCCTAGCAGCG
CTCCACCTTCTATTCCTACACGAAACAGGATCAAACAACCCCCTAGGAATCACCTCCCATTCCGACAAAATCACC
TTCCACCCCTACTACACAATCAAAGACATCCTCGGCTTATTTCTCTTTCTTCTCACCCTAATGACGTTAACACTA
TTCTCACCA
>Pse_5
CAGCTAAGGACTGCAAAACCCCACTCTGCATCAACTGAACGCAAATCAGCCACTTTAATTAAGCTAAGCCCTTA
CTAGATCAATTGGACTTAAACCCATAAACACTTAGTTAACAGCTAAACACCCTAATCAACGCTTCAATCTACTT
CTCCCGCCGCAGGAAAAAAAGGCGGGAGAAGCCCCGCCAGGTTTGAAGCTGCTTCTTCGAATTTGCAATTCAAT
ATGAAAATCACCTCAGAGCTGGTAAAAAGAGGCTTAGCCCCTGTCTTTAGATTTACAGTCCAATGCTTCACTCA
GCCATTTTACCTTATCCCACTGATGTTCGCCGACCGTTGATTGTTCTCTACAAACCACAAAGACATTGGAACAC
TGTACCTACTATTCGGCGCATGAGCTGGAGTCCTGGGCACAGCTCTAAGTCTCCTTATTTGGGCTGAGCTAGGC
CAACCAGGCAACCTTCTAGGTAACGACCACATCTACAATGTCATCGTCACAGCCCATGCATTTGTAATAATCTT
CTTCATAGTGATACCTATCATAATCGGAGGCTTTGGCAACTGACTAGTTCCCCTAAAAATCGGTGCCCCCGATA
TGGCATTCCCCCGCATAAACAACATAAGCTTCTGACTCCTACCCCCTTCTCTCCTACTTCTGCTTGCATCTGCCA
TAGTGGAAGCCGGCGCCGGAACAGGTTGGACAGTCTACCCTCCCTTAGCAGGGAACTACTCCCACCCTGGAGCT
TCCGTAGATCTAACCATCTTCTCCTTGCATCTAGCAGGCATCTCCTCTATCCTAGGAGCCATTAACTTCATTAC

AACAATCATTAATATAAAACCCCCTGCCATAACCCAATACCAAACACCCCTTTTCGTCTGATCCGTCCTAATCAC
AGCAGTCTTACTTCTCCTATCCCTCCCAGTCCTAGCTGCTGGCATCACCATGCTGTTAACAGACCATAACCTCAA
CACCACCTTCTTCGACCCAGCCAGGGGAGGGGACCCTATTCTATACCAACACCTAT
>Pse_6
CACCTCCGCTACCATAATCATCGCTATTCCCACCGGCGTCAAAGTATTTAGCTGACTCGCTACACTCCACGGAAG
CAATATGAAATGATCTGCTGCGGTGCTCTGAGCCCTGGGATTCATTTTTCTCTTCACTGTAGGCGGCCTAACTG
GCATTGTATTAGCAAACTCATCACTAGACATCGTACTACACGACACATACTACGTCGTAGCCCACTTCCACTAC
GTCCTATCAATAGGAGCTGTATTCGCCATCATAGGGGGCTTCATTCACTGATTTCCCCTATTCTCAGGCTACAC
CCTAGACCAAACCTACGCTAAAATCCATTTCGCTATCATATTCATCGGCGTAAACCTAACTTTCTTCCCACAAC
ACTTTCTTGGCCTATCTGGAATACCCCGACGTTACTCGGACTACCCCGATGCATACACCACATGAAATATCCTA
TCATCTGTAGGCTCATTCATTTCCCTAACAGCAGTAATATTAATAATTTTCATGATTTGAGAAGCCTTCGCTTC
AAAGCGAAAAGTCCTAATAATAGAAGAACCCTCCATAAACCTGGAGTGACTATATGGATGCGCCCCACCCTACC
ACACATTCGAAGAACCCGTATACATAAAATCTAGACAAAAAAGGAAGGAATCGAACCCCC
>Pse_7
GTTTATGTAGCTTACTTCCTCAAAGCAATACACTGAAAATGTTTCGACAGGCTCATACCACCCCATAAACAAAT
AGGTTTGGTCGTAGCCTATTAGCTCTTAGTAAGATTACACATGCAAGCATCCCTGCCCCGGTGAGTCACCCTCT
AAATCACCACGATCAAAAGGAACAAGTATCAAGCACGCAGCAACGCAGCTCAAAACGCTTAGCCTAGCCACACC
CCCACGGGAGACAGCAGTGATAAACCTTTAGCAATAAACGAAAGTTTAACTAAGCTATACTAACCCTAGGGTTC
GTCAAGTTCGTGCCAGCCACCGCGGTCACACGATTAACCCAAGTCAATAGAAACCGGCGTAAAGAGTGTTTTAG
ATCAATTCCCCTCAATAAAGCTAAAATTCACCTGAGTTGTAAAAAACTCCAGTTGATACAAAATAAACTACGAA
AGTGGCTTTAACGCATCTGAACACACAATAGCTAAGACCCAAACTGGGATTAGATACCCCACTATGCTTAGCCC
TAAACTTCAACAGTTAAATTAACAAAACTGCTCGCCAGAACACTACGAGCCACAGCTTAAAACTCAAAGGACCT
GGCGGTGCTTCATATCCCTCTAGAGGAGCCTGTTCTGTAATCGATAAACCCCGATCAACCTCACCACCTCTTGC
TCAGCCTATATACCGCCATCTTCAGCAAACCCTGACGAAGGCTGCAAAGTAAGCGCAAGTACCCACGTAAAGAT
GTTAGGTCAAGGTGTAGCCCATGAGCTGGCAAGAAATGGGCTACATTTTCTACTTCAGAAAACTACGATAACCC
TTATGAAATTTAAGGGTCGAAGGTGGATTTAGCAGTAAACTAAGAGTAGAGTGCGTAGTTGAACAGGGCCCTG
AAGCGCGTACACACCGCCCGTCACCCTCCTCAAGTATACTTCAAAGGACATTTAACTAAAACCCCTGCGCATTT
ATATAGAGGAGATAAGTCGTAACATGGTAAGTGTACTGGAAAGTGCACTTGGACGAACCAGAGTGTAGCTTAA
CATAAAGCACCCAACTTACACTTAGGAGATTTCAACTCAACTTGACCACTCTGAGCTAAACCTAGCCCCAAACC
CATTCCACCTTACTACCAAACAACCTTAACCAAACCATTTACTCAAATAAAGTATAGGCGATAGAAATTGTAAA
CCGGCGCAATAGATATAGTACCGCAAGGGAAAGATGAAAAATTATAACCAAGCATAATATAGCAAGGACTAAC
CCCTGTACCTTCTGCATAATGAATTAACTAGAAATAACTTTGCAAAGAGAACCAAAGCTAAGACCCCCGAAACC
AGACGAGCTACCTAAGAACAGCTAAAAGAGCATACCCGTCTATGTAGCAAAATAGTGGGAAGATTCATAGGTA
GAGGCGACAAACCTACCGAGTCTGGTGATAGCTGGTTGTCCAAGATAGAATCTTAGTTCAACTTTAAATTTACC
CACAGAACCCTCCAAATCCCCCTGTAAATTTAACTGTTAGTCCAAAGAGGAACAGCTCTTTGGACACTAGGAAA
AAACCTTGTAAAGAGAGTAAAAAATTTAATACCCATAGTAGGCCTAAAAGCAGCCACCAATTAAGAAAGCGTT
CAAGCTCAACACCTATCATCCGAAAAATCCCAAACATATGACTGAACTCCTTGCACCCAATTGGACCAATCTAT
TACCTTATAGAAGAACTAACGTTGGTATAAGTAACATGAAAGCATTCTCCTCCACATAAGCCTACATCAGACCA
AAATATTAAACTGACAATTAACAGCCTAATATCTACAACCAACCAACAAGCCATTATTACCCCCACTGTTAACC
CAACACAGGCATGCCCACAAGGAAAGGTTAAAAAAAGTAAAAGGAACTCGGCAAACCTTACCCCACCTGTTTAC
CAAAAACATCACCTCTAGCATTATCAGTATTAGAGGCACCCCTGCCCGGTGACATATGTTTAACGGCCGCGGTA
CCCTAACCGTGCAAAGGTAGCATAATCACTTGTTCCTTAAATAGGGACTTGTATGAATGGCTCCACGAGGGTTC
AGCTGTCTCTTACTTTCAACCAGTGAAATTGACCTGCCCGTGAAGAG

>Pse_8
TTCACTGATTTCCCCTATTCTCAGGCTACACCTTAGACCAAACCTACGCCAAAATCCATTTTGCTATCATATTC
GTCGGCGTAAACCTAACTTTCTTCCCACAACACTTTCTCGGCCTATCCGGAATGCCCCGACGCTACTCGGACTAC
CCCGATGCATACACCACATGAAACATCCTATCATCTGTAGGCTCATTCATTTCTCTAACAGCAGTAGTATTAAT
AATTTTCATGATTTGAGAGGCCTTCGCTTCGAAGCGAAAAGTCCTAATAGTAGAAGAGCCCTCCATAAACCTGG
AGTGACTATATGGATGCCCCCCACCCTACCACACATTCGAAGAACCCGTATACATAAAATCTAGACAAAAAAGG
AAGGAATCGAACCCCCCAGAGCTGGTTTCAAGCCAACCCCATGGCCTCCATGACTTTTTCAAAAAGATATTAGA
AAAACCATTTCATAACTTTGTCAAAGTTAAGTTATAGGCTAAATCCTATATATCTTAATGGCACATGCAGCGCA
AGTAGGTTTACAAGACGCTACTTCCCCTATCATAGAAGAGCTTATCATCTTTCATGATCACGCCCTCATAATCA
TTTTCCTTATCTGCTTCCTAGTCCTGTACGCCCTTTTCCTAACACTCACAACAAAACTAACTAATACTAACATC
TCAGACGCTCAGGAAATAGAAACCGTCTGAACTATCCTGCCCGCCATCATCCTAGTCCTTATCGCCCTCCCATCC
CTACGCATCCTTTACATAACAGACGAGGTCAACGACCCCTCCTTTACCATCAAATCAATTGGCCATCAATGGTA
TTGAACCTACGAATACACCGACTACGGTGGACTAATCTTCAACTCCTACATACTTCCCCCATTATTCCTAGAAC
CAGGCGACCTGCGACTCCTTGACGTTGACAATCGAGTGGTACTCCCGGTTGAAGCCCCCATTCGCATAATAATT
ACATCACAAGATGTCTTACACTCATGAGCTGTTCCCACATTAGGCCTAAAAACAGATGCAATTCCCGGACGCCT
GAACCAAACCACTTTCACTGCTACACGACCAGGGGTGTACTACGGCCAATGCTCTGAAATCTGTGGAGCAAACC
ACAGTTTTATACCCATCGTCCTAGAATTAATTCCCCTAAAAATCTTTGAAATAGGACCCGTATTTACCCTATAG
CACCTTCTCTACCCCCTCTAGAGCCCACTGTAAAGCTAACTTAGCATTAACCTTTTAAGTTAAAGATTAAGAGA
ACCAACACCTCTTTACAGTGAAATGCCCCAACTAAATACTGCCGTATGGCCCACCATAATTGCCCCCATACTCCT
CACACTATTCCTCATCACCCAACTAAAAATATTAAATACAAATTACCATCTACCTCCCTCACMAAGCCCATAAA
AA
>Pse_9
AAGGGAGTCCGAACTAGTCTCAGGCTTCAACATCGAATACGCCGCAGGCCCCTTCGCCCTATTCTTCATAGCCG
AATACACAAACATTATTATAATAAACACCCTCACCACTACAATCTTCCTAGGAACAACATATAACGCACTCTCC
CCTGAACTCTACACAACATATTTGTCACCAAGACCCTACTTCTGACCTCCCTGTTCTTATGAATTCGAACAGC
ATACCCCCGATTCCGCTACGACCAACTCATACACCTCCTATGAAAAAACTTCCTACCACTCACCCTAGCATTACT
TATATGATATGTCTCCATACCCATTACAATCTCCAGCATTCCCCCTCAAACCTAAGAAATATGTCTGATAAAAG
AGTTACTTTGATAGAGTAAATAATAGGAGTTTAAATCCCCTTATTTCTAGGACTATGAGAATCGAACCCATCCC
TGAGAATCCAAAATTCTCCGTGCCACCTATCACACCCCATCCTAAAGTAAGGTCAGCTAAATAAGCTATCGGGC
CCATACCCCGAAAATGTTGGTTATATCCTTCCCGTACTAATTAATCCCCTGGCCCAACCCGTCATCTACTCTACC
ATCTTTGCAGGCACACTCATCACAGCGCTAAGCTCGCACTGATTTTTTACCTGAGTAGGCCTAGAAATAAACAT
GCTAGCTTTTATTCCAGTTCTAACCAAAAAAATAAACCCTCGTTCCACAGAAGCTGCCATCAAGTATTTCCTCA
CGCAAGCAACCGCATCCATAATCCTTCTAATAGCTATCCTCTTCAACAATATACTCTCCGGACAATGAACCATA
ACCAATACCACCAATCAATACTCATCATTAATAATCATAATGGCTATAGCAATAAAACTAGGAATAGCCCCCTT
TCACTTCTGAGTCCCAGAGGTTACCCAAGGCACCCCTCTGACATCCGGCCTGCTCCTTCTCACATGACAAAAACT
AGCCCCCATCTCAATCATATACCAAATTTCTCCCTCATTAAACGTAAGCCTTCTCCTCACTCTTTCAATCTTATC
CATCATGGCAGGCAGTTGAGGTGGATTAAACCAAACCCAACTACGCAAAATCTTAGCATACTCCTCAATTACCC
ACATAGGATGAATAACAGCAGTTCTACCGTACAACCCTAACATAACCATTCTTAATTTAACTATTTATATTATC
CTAACTACTACCGCATTCCTACTACTCAACTTAAACTCCAGCACCACAACCCTACTACTATCTCGCACCTGAAAC
AAGCTAACATGACTAACACCCTTAATTCCATCCACCCTCCTCTCCCTAGGAGGCCTGCCCCCGCTAACCGGCTTT
TTGCCCAAATGGGCCATTATCGAAGAATTCACAAAAAACAATAGCCTCATCATCCCCACCATCATAGCCATCAT
CACCCTCCTTAACCTCTACTTCTACCTGCGCCTAATCTACTCCACCTCAATCACACTACTCCCTATATCTAACAA
CGTAAAAATAAAATGACAGTTTGAACACACAAAACCCACCCCATTCCTCCCCACACTCATCGCCCTTACCACACT

GCTCCTACCTATCTCCCCTTTTATGCTAATAATCTTATAGAAATTTAGGTTAAATACAGACCAAGAGCCTTCAA
AGCCCTCAGTAAGTTGCAATACTTAATTTCTGCAACAGCTAAGGACTGCAAAACCCCACTCTGCATCAACTGAA
CGCAAATCAGCCACTTTAATTAAGCTAAGCCCTTACTAGACCAATGGGACTTAAACCCACAAACACTTAGTTAA
CAGCTAAGCACCCTAATCAACTGGCTTCAATCTACTTCTCCCGCCGCCGGGAAAAAAGGCGGGAGAAGCCCCGG
CAGGTTTGAAGCTGCTTCTTCGAATTTGCAATTCAATATGAAAATCACCTCAGAGCTGGTAAAAAGAGGCTTA
ACCCCTGTCTTTAGATTTACAGTCCAATGCTTCACTCAGCCATTTTACCTCACCCCCACTGATGTTCGCCGACCG
TTGACTATTCTCTACAAACCACAAAGACATTGGAACACTATACCTATTATTCGGCGCATGAGCTGGAGTCCTAG
GCACAGCTCTAAGCCTCCTTATTCGAGCCGAACTGGGCCAGCCAGGCAACCTTCTAGGTAACGACCACATCTAC
AACGTTATCGTCACAGCCCATGCATTTGTAATAATCTTCTTCATAGTAATACCCATCATAATCGGAGGCTTTGG
CAACTGACTAGTTCCCCTAATAATCGGTGCCCCCGATATGGCGTTTCCCCGCATAAACAACATAAGCTTCTGAC
TCTTACCCCCCTCTCTCCTACTCCTGCTTGCATCTGCTATAGTGGAGGCCGGCGCAGGAACAGGTTGAACAGTC
TACCCTCCCTTGGCAGGGAACTACTCCCACCCTGGAGCCTCCGTAGACCTAACCATCTTCTCCTTACACCTAGCA
GGTATCTCCTCTATCTTAGGAGCCATCAATTTCATCACAACAATTATTAATATAAAACCCCCTGCCATAACCCA
ATACCAAACGCCCCTTTTCGTCTGATCCGTCCTAATCACAGCAGTCTTACTTCTCCTATCTCTCCCAGTCCTAGC
CGCTGGCATCACTATACTACTAACAGACCGTAACCTCAACACCACCTTCTTCGACCCAGCCGGAGGAGGAGACCC
CATTCTATACCAACACCTATTCTGATTTTTCGGTCACCCTGAAGTTTATATTCTCATCCTACCAGGCTTCGGAA
TAATCTCCCATATTGTAACTTACTACTCCGGGAAAAAAAGAACCATTTGGATACATAGGTATGGTCTGAGCTAT
GATATCAATTGGCTTCCTAGGGTTTATCGTGTGAGCACACCATATATTTACAGTAGGAATAGACGTAGACACAC
GAGCATATTTCACCTCCGCTACCATAATCATCGCTATCCCCACCGGCGTCAAAGTATTTAGCTGACTCGCCACAC
TCCACGGAAGCAATATGAAATGATCTGCTGCAGTGCTCTGAGCCCTAGGATTTATTTTTCTTTTCACCGTAGGT
GGCCTGACTGGCATTGTATTAGCAAACTCATCACTAGACATCGTACTACACGACACGTACTACGTTGTAGCCCA
CTTCCACTATGTCCTATCAATAGGAGCTGTATTTGCCATCATAGGAGGCTTCATTCACTGATTTCCCCTATTCT
CAGGCTACACCCTAGACCAAACCTACGCCAAAATCCATTTCGCTATCATATTCATCGGCGTAAATCTAACTTTC
TTCCCACAACACTTTCTCGGCCTATCCGGAATGCCCCGACGTTACTCGGACTATCCCGATGCATACACCACATGA
AATATCCTATCATCTGTAGGCTCATTCATTTCTCTAACAGCAGTAATATTAATAATTTTCATAATTTGAGAAGC
CTTCGCTTCGAAGCGAAAAGTCCTAATAGTAGAAGAACCCTCCATAAACCTGGAGTGACTATATGGATGCCCCC
CACCCTACCACACATTCGAAGAACCCGTATACATAAAATCTAGACAAAAAAGGAAGGAATCGAACCCCCCAAAG
CTGGTTTCAAGCCAACCCCATGGCCTCCATGACTTTTTCAAAAAGATATTAGAAAAACCATTTCATAACTTTGT
CAAAGTTAAATTATAGGCTAAATCCTATATATCTTAATGGCACATGCAGCGCAAGTAGGTCTACAAGACGCTAC
TTCCCCTATCATAGAAGAGCTTATCATCTTTCATGATCACGCCCTCATAATCATTTTCCTTATCTGCTTCCTAG
TCCTGTACGCCCTTTTCCTAACACTCACAACAAAACTAACTAATACTAACATCTCAGACGCTCAGGAAATAGAA
ACCGTCTGAACTATCCTGCCCGCCATCATCCTAGTCCTTATCGCCCTCCCATCCCTACGCATCCTTTACATAACA
GACGAGGTCAACGATCCCTCCTTTACCATCAAATCAATTGGCCATCAATGGTACTGAACCTACGAATACACCGA
CTACGGCGGACTAATCTTCAACTCCTACATACTTCCCCCATTATTCCTAGAACCAGGCGACCTGCGACTCCTTGA
CGTTGACAATCGAGTAGTACTCCCGGTTGAAGCCCCCATTCGTATAATAATTACATCACAAGACGTCTTACACT
CATGAGCTGTCCCCACATTAGGCTTAAAAACAGATGCAATTCCCGGACGTCTAAACCAAACCACTTTCACTGCT
ACACGACCAGGGGTATACTACGGCCAATGCTCTGAAATCTGTGGAGCAAACCAGTTTTATGCCCATCGTCCTAG
AATTAATTCCCCTAAAAATCTTTGAAATAGGGCCCGTATTTACCCTATAGCACCCCCTCTACCCCCTCTAGAGCC
CACTGTAAAGCTAACTTAGCATTAACCTTTTAAGTTAAAGATTAAGAGAACCAACACCTCTTTACAGTGAAATG
CCCCAACTAAATACTACCGTATGACCCACCATAATTACCCCCATACTCCTTACACTATTCCTCATCACCCAACTA
AAAATATTAAATACAAATTACCACCTACCTCCCTCACCAAAGCCCATAAAAATAAAAAACTATAACAAACCCTG
AGAACCAAAATGAACGAAAATCTGTTCACTTCATTCATTGCCCCCACAATCCTAGGCCTACCCGCCGCAGTACT
GATCATTCTATTTCCCCCTCTATTGATCCCCACCTCCAAATATCTCATCAACAACCGACTAATTACCACCCAACA

ATGACTAATCCAACTAACCTCAAAACAAATGATAGCCATACACAACACTAAGGGACGAACCTGATCTCTTATAC
TAGTATCCTTAATCATTTTTATTGCCACAACTAACCTCCTCGGACTCCTGCCTCACTCATTTACACCAACCACCC
AACTATCTATAAACCTAGCCATGGCCATCCCCTTATGAGCGGGCGCAGTGATTATAGGCTTTCGCTCTAAGATT
AAAAATGCCCTAGCCCACTTCTTACCACAAGGCACACCTACACCCCTTATCCCTATACTAGTTATTATCGAAACC
ATCAGCCTACTCATTCAACCAATAGCCCTGGCCGTACGCCTAACCGCTAACATTACTGCAGGCCACCTACTCATG
CACCTAATTGGAAGCGCCACACTAGCAATATCAACTATTAACCTTCCCTCTACACTTATCATCTTCACAATTCT
AATTCTACTGACTATCCTAGAAATCGCTGTCGCCTTAATCCAAGCCTACGTTTTTACACTTCTAGTAAGCCTCT
ACCTGCACGACAACACATAATGACCCACCAATCACATGCCTATCATATAGTAAAACCCAGCCCATGGCCCCTAA
CAGGGGCCCTCTCAGCCCTCCTAATGACCTCCGGCCTAGCCATGTGATTTCACTTCCACTCCACAACCCTCCTCA
TACTAGGCCTACTAACCAACACACTAACCATATACCAATGATGGCGCGATGTAACACGAGAAAGCACATACCAA
GGCCACCACACACCACCTGTCCAGAAAGGCCTTCGATACGGGATAATCCTATTTATTACCTCAGAAGTTTTTT
CTTCGCAGGATTTTTCTGAGCCTTTTACCACTCCAGCCTAGCTCCCACCCCCCAACTAGGGGGACACTGGCCCCC
AACAGGCATCACCCCGCTAAATCCCCTAGAAGTCCCACTCCTAAACACATCCGTATTACTCGCATCAGGGGTAT
CAATCACCTGAGCTCACCATAGTCTAATAGAAAACAACCGAAACCAAATAATTCAAGCACTGCTTATTACAATT
TTACTGGGTCTCTATTTTACCCTCCTACAAGCCTCAGAGTACTTCGAG

>Pse_10

TTCATACACCTATCCCCCATTCTCCTCCTATCCCTCAACCCCGACATCATYAYCGGGTTTTCCTCTTGTAAATAT
AGTTTAACCAAAACATCAGATTGTGAATCYGACAACAGAGGCTYACGACCCCTTATTTACCGAGAAAGCTCACA
AGAACTGCTAACTCATGCCCCCATGTCTAACAACATGGCTTTCTCAACTTTTAAAGGATAACAGCTATCCATTG
GTCTTAGGCCCCAAAAATTTTGGTGCAACTCCAAATAAAAGTAATAACCATGCACACTACTATAACCACCCTAA
CCCTGACTTCCCTAATTCCCCCCATCCTTACCACCCTCGTTAACCCTAACAAAAAAAACTCATACCCCCATTATG
TAAAATCCATTGTCGCATCCACCTTTATTATCAGTCTCTTCCCCACAACAATATTCATGTGCCTAGACCAAGAA
GTTATTATCTCGAACTGACACTGAGCCACAACCCAAACAACCCAGCTCTCCCTAAGCTTCAAACTAGACTACTT
CTCCATAATATTCATCCCTGTRGCATTGTTCGTTACATGRTCCATCATAGAATTCTCACTGTGATATATATAAACT
CAGACCCAAACATTAATCAGTTCTTCAAATATCTACTCATTTTCCTAATTACCATACTAATCTTAGTTACCGCT
AACAACCTATTCCAACTGTTCATCGGCTGAGAGGGCGTAGGAATTATATCCTTCTTGCTCATCAGTTGATGATA
CGCCCGAGCAGATGCCAACACAGCAGCCATTCAAGCARTCCTATACAACCGTATCGGCGATATCGGTTTCATCC
TCGCCTTAGCATGATTTATCCTACACTCCAACTCATGAGACCCACAACAAATAGCCCTTCTAAACGCTAATCCA
AGCCTCACCCCACTACTAGGCCTCCTCCTAGCAGCAGCAGGCAAATCAGCCCAATTAGGCCTCCACCCCTGACTC
CCCTCAGCCATAGAAGGCCCCACCCCGGTCTCAGCCCTACTCCACTCAAGCACTATAGTCGTAGCAGGAGTCTTC
TTACTCATCCGCTTCCACCCCCTAGCAGAAAATAGCCCACTATCCAACTCWACGACTATGCTTAGGCGCTATCA
CCACTCTGTTCGCAGCAGTCTGCGCCCTTACACAAAATGACATCAAAAAAATCGTAGCCTTCTCCACTTCAAGT
CAACTAGGRCTCATARTAGTTACAATCGGCATCAACCAACCACACCTAGCATTCCTGCACATCTGTACCCACGC
CTTCTTCAAAGCCATACTATTTATRTGCTCCGGGTCCATCATCCACAACCTTAAACAATGAACAAGATATTCGA
AAAATAGGAGGACTACTCAAAACCATACCTCTCACTTCAACCTCCCTCACCATTGGCAGCCTAGCATTAGCAGG
AATACCTTTCCTCACAGGTTTCTAYTCCAAAGACCACATCATYGAAACCGCAAACATATCATACACAAACGCCT
GAGCCCTATCTATTACTCTCATCGCTACCTCCCTGACAAGCGCCTATAGCACTCGAATAATTCTTCTCACCCTAA
CAGGTCAACCTCGCTTCCCYACCCTYACTAACATTAACGAAAATAACCCYACCCTACTAAACCCCATTAAACGCC
TGGCAGCCGGAAGCCTATTCGCAGGATTTCTT

>Pse_11

AGCTGCCATCAAGTATTTCCTCACGCAAGCAACCGCATCCATAATCCTTCTAATAGCTATCCTCTTCAACAATA
TACTCTCCGGACAATGAACCATAACCAATACTACCAATCAATACTCATCATTAATAATCATAATGGCTATAGCA
ATAAAACTAGGAATAGCCCCCTTTCACTTCTGAGTCCCAGAGGTTACCCAAGGCACCCCTCTGACATCCGGCCT

GCTTCTTCTCACATGACAAAAACTAGCCCCCATCTCAATCATATACCAAATCTCTCCCTCACTAAACGTAAGCCT
TCTCCTCACTCTCTCAATCTTATCCATCATAGCAGGCAGTTGAGGTGGATTAAACCAAACCCAGCTACGCAAAA
TCTTAGCATACTCCTCAATTACCCACATAGGATGAATAATAGCAGTTCTACCGTACAACCCTAACATAACCATC
CTTAATTTAACTATTTATATTATCCTAACTACTACCGCATTCCTACTACTCAACTTAAACTCCAGCACCACGAC
CCTACTACTATCTCGCACCTGAAACAAGCTAACATGACTAACACCCTTAATTCCATCCACCCTCCTCTCCCTAGG
AGGCCTGCCCCCGCTAACCGGCTTTTTGCCCAAATGGGCCATTATCGAAGAATTCACAAAAAACAATAGCCTCA
TCATCCCCACCATCATAGCCACCATCACCCTCCTTAACCTCTACTTCTACCTACGCCTAATCTACTCCACCTCAA
TCACACTACTCCCCATATCTAACAACGTAAAAATAAAATGACAGTTTGAACATACAAAACCCACCCCATTCCTC
CCCACACTCATCACCCTTACCACGCTACTCCTACCTATCTCCCCTTTTATACTAATAATCTTATAGAAATTTAGG
TTAAATACAGACCAAGAGCCTTCAAAGCCCTCAGTAAGTTGCAATACTTAATTTCTGTAACAGCTAAGGACTGC
AAAACCCCACTCTGCATCAACTGAACGCAAATCAGCCACTTTAATTAAGCTAAGCCCTTACTAGACCAATGGGA
CTTAAACCCACAAACACTTAGTTAACAGCTAAGCACCCTAATCAACTGGCTTCAATCTACTTCTCCCGCCGCCGG
GAAAAAAGGCGGGAGAAGCCCCGGCAGGTTTGAAGCTGCTTCTTCGAATTTGCAATTCAATATGAAAATCACCT
CGGAGCTGGTAAAAAGAGGCCTAACCCCTGTCTTTAGATTTACAGTCCAATGCTTCACTCAGCCATTTTACCTC
ACCCCCACTGATGTTCGCCGACCGTTGACTATTCTCTACAAACCACAAAGACATTGGAACACTATACCTATTAT
TCGGCGCATGAGCTGGAGTCCTAGGCACAGCTCTAAGCCTCCTTATTCGAGCCGAGCTAGGCCAGCCAGGCAAC
CTTCTAGGTAACGACCACATCTACAACGTTATCGTCACAGCCCATGCATTTGTAATAATCTTCTTCATAGTAAT
ACCCATCATAATCGGAGGCTTTGGCAACTGACTAGTTCCCCTAATAATCGGTGCCCCCGATATGGCGTTTCCCC
GCATAAACAACATAAGCTTCTGACTCTTACCTCCCTCTCTCCTACTCCTGCTCGCATCTGCTATAGTGGAGGCCG
GAGCAGGAACAGGTTGAACAGTCTACCCTCCCTTAGCAGGGAACTACTCCCACCCTGGAGCCTCCGTAGACCTA
ACCATCTTCTCCTTACACCTAGCAGGTGTCTCCTCTATCTTAGGGGCCATCAATTTCATCACAACAATTATTAA
TATAAAACCCCCTGCCATAACCCAATACCAAACGCCCCTTTTCGTCTGATCCGTCCTAATCACAGCAGTCCTACT
TCTCCTATCTCTCCCAGTCCTAGCTGCTGGCATCACTATACTACTAACAGACCGCAACCTCAACACCACCTTCTT
CGACCCCGCCGGAGGAGGAGACCCCATTCTATACCAGCACCTATTCTGATTTTTCGGTCACCCTGAAGTTTATA
TTCTCATCCTACCAGGCTTCGGAATAATCTCCCATATTGTAACTTACTACTCCGGAAAAAAAGAACCATTTGGA
TACATAGGTATGGTCTGAGCTATGATATCAATTGGCTTCCTAGGGTTTATCGTGTGAGCACACCATATATTTAC
AGTAGGAATAGACGTAGACACACGAGCATATTTCACCTCCGCTACCATAATCATCGCTATCCCCACCGGCGTCA
AAGTATTTAGCTGACTCGCCACACTCCACGGAAGCAATATGAAATGATCTGCTGCAGTGCTCTGAGCCCTAGGA
TTCATCTTTCTTTTCACCGTAGGTGGCCTGACTGGCATTGTATTAGCAAACTCATCACTAGACATCGTACTACA
CGACACGTACTACGTTGTAGCTCACTTCCACTATGTCCTATCAATAGGAGCTGTATTTGCCATCATAGGAGGCT
TCATTCACTGATTTCCCCTATTCTCAGGCTACACCCTAGACCAAACCTACGCCAAAATCCATTTCGCTATCATAT
TCATCGGCGTAAATCTAACTTTCTTCCCACAACACTTTCTCGGCCTATCCGGAATGCCCCGACGTTACTCGGACT
ACCCCGATGCATACACCACATGAAATATCCTATCATCTGTAGGCTCACTCATTTCTCTAACAGCAGTAATATTA
ATAATTTTCATGATTTGAGAAGCCTTCGCTTCGAAGCGAAAAGTCCTAATAGTAGAAGAACCCTCCATAAACCT
GGAGTGACTATATGGATGCCCCCCACCCTACCACACATTCGAAGAACCCGTATACATAAAATCTAGACAAAAAA
GGAAGGAATCGAACCCCCCAAAGCTGGTTTCAAGCCAACCCCATGGCCTCCATGACTTTTTCAAAAAGATATTA
GAAAAACCATTTCATAACTTTGTCAAAGTTAAATTATAGGCTAGATCCTATATATCTTAATGGCACATGCAGCG
CAAGTAGGTCTACAAGACGCTACTTCCCCTATCATAGAAGAGCTTATCACCTTTCATGATCACGCCCTCATAAT
CATTTTCCTTATCTGCTTCCTAGTCCTGTATGCCCTTTTCCTAACACTCACAACAAAACTAACTAATACTAACA
TCTCAGACGCTCAGGAAATAGAAACCGTCTGAACTATCCTGCCCGCCATCATCCTAGTCCTCATCGCCCTCCCAT
CCCTACGCATCCTTTACATAACAGACGAGGTCAACGATCCCTCCTTTACCATCAAATCAATTGGCCACCAATGG
TACTGAACCTACGAGTACACCGACTACGGCGGACTAATCTTCAACTCCTACATACTTCCCCCATTATTCCTAGA
ACCAGGCGACCTGCGACTCCTTGACGTTGACAATCGAGTAGTACTCCCGATTGAAGCCCCCATTCGTATAATAA

*TTACATCACAAGACGTCTTACACTCATGAGCTGTCCCCACATTAGGCTTAAAAACAGATGCAATTCCCGGACGT
CTAAACCAAACCACTTTCACCGCTACACGACCGGGGGTATACTACGGTCAATGCTCTGAAATCTGTGGAGCAAA
CCACAGTTTCATGCCCATCGTCCTAGAATTAATTCCCCTAAAAATCTTTGAAATAGGGCCCGTATTTACCCTAT
AGCACCCCCTCTACCCCCTCTAGAGCCCACTGTAAAGCTAACTTAGCATTAACCTTTTAAGTTAAAGATTAAGA
GAACCAACACCTCTTTACAGTGAAATGCCCCAACTAAATACTACCGTATGGCCCACCATAATTACCCCCATACT
CCTTACACTATTCCTCATCACCCAACTAAAAATATTAAACACAAATTACCACTTACCTCCCTCACCAAAGCCCAT
AAAAATAAAAAATTATAACAAACCCTGAGAACCAAAATGAACGAAAATCTGTTCGCTTCATTCATTGCCCCCAC
AATCCTAGGCCTACCCGCCGCAGTACTGATCATTCTATTTCCCCCTCTATTGATCCCCACCTCCAAATATCTCAT
CAACAACCGACTAATTACCACCCAACAATGACTAATCAAACTAACCTCAAAACAAATGATAGCCATACACAACA
CTAAGGGACGAACCTGATCTCTTATACTAGTATCCTTAATCATTTTTATTGCCACAACTAACCTCCTCGGACTC
CTACCTCACTCATTTACACCAACCACCCAACTATCTATAAACCTAGCCATGGCCATCCCCTTATGAGCGGGCGCA
GTGATTATAGGCTTTCGCTCTAAGATTAAAAATGCCCTAGCCCACTTCTTACCACAAGGCACACCTACACCCCT
TATCCCCATACTAGTTATTATCGAAACCATCAGCCTACTCATTCAACCAATAGCCCTGGCCGTACGCCTAACCGC
TAACATTACTGCAGGCCACCTACTCATGCACCTAATTGGAAGCGCCACCCTAGCAATATCAACCATTAACCTTC
CCTCTACACTTATCATCTTCACAATTCTAATTCTACTGACTATCCTAGAAATCGCTGTCGCCTTAATCCAAGCC
TACGTTTTCACACTCCTAGTAAGCCTCTACCTGCACGACAACACATAATGACCCACCAATCACATGCCTATCAT
ATAGTAAAACCCAGCCCATGACCCCTAACAGGGGCCCTCTCAGCCCTCCTAATGACCTCCGGCCTAGCCATGTGA
TTTCACTTCCACTCCACAACGCTCCTCATACTAGGCCTACTAACCAACACACTAACCATATACCAATGATGGCGC
GATGTAACACGAGAAAGCACATACCAAGGCCACCACACACCACCTGTCCAAAAAGGCCTTCGATACGGGATAAT
CCTATTTATTACCTCAGAAGTTTTTTTCTTCGCAGGATTTTTCTGAGCCTTTTACCACTCCAGCCTAGCCCCTA
CCCCCCAACTAGGAGGGCACTGGCCCCCAACAGGCATCACCCCGCTAAATCCCCTAGAAGTCCCACTCCTAAACA
CATCCGTATTACTCGCATCAGGAGTATCAATCACCTGAGCTCACCATAGTCTAATAGAAAACAACCGAAACCAA
ATAATTCAAGCACTGCTTATTACAATTTTACTGGGTCTCTATTTTACCCTCCTACAAGCCTCAGAGTACTTCGA
ATCTCCCTTCACCATTTCCGACGGCATCTACGGCTCAACATTTTTTGTAGCCACAGGCTTCCACGGACTTCACGT
CATTATTGGCTCAACTTTCCTCACTATCTGCTTCATCCGCCAACTAATATTTCACTTTACATCCAAACATCACT
TTGGCTTCGAAGCCGCCGCCTGATACTGGCATTTTGTAGATGTGGTTTGACTATTTCTGTATGTCTCCATCTAT
TGATGAGGGTCTTACTCTTTTAGTATAAATAGTACCGTTAACTTCCAATTAACTAGTTTTGACAACATTCAAAA
AAGAGTAATAAACTTCGCCTTAATTTTAATAATCAACACCCTCCTAGCCTTACTACTAATAATTATTACATTTT
GACTACCACAACTCAACGGCTACATAGAAAAATCCACCCCTTACGAGTGCGGCTTCGACCCTATATCCCCCGCCC
GCGTCCCTTTCTCCATAAAATTCTTCTTAGTAGCTATTACCTTCTTATTATTTGATCTAGAAATTGCCCTCCTT
TTACCCCTACCATGAGCCCTACAAACAACTAACCTGCCACTAATAGTTATGTCATCCCTCTTATTAATCATCAT
CCTAGCCCTAAGTCTGGCCTATGAGTGACTACAAAAAGGATTAGACTGAGCCGAATTGGTATATAGTTTAAACA
AAACGAATGATTTCGACTCATTAAATTATGATAATCATATTTACCAAATGCCCCTCATTTACATAAATATTATA
CTAGCATTTACCATCTCACTTCTAGGAATACTAATATATCGCTCACACCTCATATCCTCCCTACTATGCCTAGA
AGGAATAATACTATCGCTGTTCATTATAGCTACTCTCATAACCCTCAACACCCACTCCCTCTTAGCCAATATTG
TGCCTATTGCCATACTAGTTTTTGCCGCCTGCGAAGCAGCGGTAGGCCTAGCCCTACTAGTCTCAATCTCCAAC
ACATATGGCCTAGACTACGTACATAACCTAAACCTGCTCCAATGCTAAAACTAATCGTCCCAACAATTATATTA
CTACCACTGACATGACTCTCCAAAAAACACATAATTTGAATCAACACAACCACCCACAGCCTAATTATTAGCAT
CATCCCCCTACTATTTTTTAACCAAATCAACAACAACCTATTTAGCTGCTCCCCAACCTTTTCCTCCGACCCCCT
AACAACCCCCCTCCTAATACTAACTACCTGACTCCTACCCCTCACAATCATGGCAAGCCAACGCCACTTATCCAG
TGAACCACTATCACGAAAAAAACTCTACCTCTCTATACTAATCTCCCTACAAATCTCCTTAATTATAACATTCA
CAGCCACAGAACTAATCATATTTTATATCTTCTTTGAAACCACACTTATCCCCACCTTGGCTATCATCACCCGA
TGAGGCAACCAGCCAGAACGCCTGAACGCAGGCACATACTTCCTATTCTACACCCTAGTAGGCTCCCTTCCCCTA*

CTCATCGCACTAATTTACACTCACAACACCCTAGGCTCACTAAACATTCTACTACTCACTCTCACTGCCCAAGAA
CTATCAAACTCCTGAGCCAACAACTTAATATGACTAGCTTACACAATAGCTTTTATAGTAAAGATACCTCTTTA
CGGACTCCACTTATGACTCCCTAAAGCCCATGTCGAAGCCCCCATCGCTGGGTCAATAGTACTTGCCGCAGTAC
TCTTAAAACTAGGCGGCTATGGTATAATACGCCTCACACTCATTCTCAACCCCCTGACAAAACACATAGCCTAC
CCCTTCCTTGTACTATCCCTATGAGGCATAATTATAACAAGCTCCATCTGCCTACGACAAACAGACCTAAAATC
GCTCATTGCATATTCTTCAATCAGCCACATAGCCCTCGTAGTAACAGCCATTCTCATCCAAACCCCCTGAAGCTT
CACCGGCGCAGTCATTCTCATAATCGCCCACGGACTTACATCCTCATTACTATTCTGCCTAGCAAACTCAAACT
ACGAACGCACTCACAGTCGCATCATAATCCTCTCTCAAGGACTTCAAACTCTACTCCCACTAATAGCTTTTTGA
TGACTTCTAGCAAGCCTCGCTAACCTCGCCTTACCCCCCACTATTAACCTACTGGGAGAACTCTCTGTGCTAGTA
ACCACATTCTCCTGATCAAATATCACTCTCCTACTTACAGGACTCAACATACTAGTCACAGCCCTATACTCCCTC
TACATATTTACCACAACACAATGGGGCTCACTCACCCACCACATTAACAACATAAAACCCTCATTCACACGAGA
AAACACCCTCATGTTCATACACCTATCCCCCATTCTCCTCCTATCCCTCAACCCCGACATCATTACCGGGTTTTC
CTCTTGTAAATATAGTTTAACCAAAACATCAGATTGTGAATCTGACAACAGAGGCTCACGACCCCTTATTTACC
GAGAAAGCTCACAAGAACTGCTAACTCATGCCCCCATGTCTAACAACATGGCTTTCTCAACTTTTAAAGGATAA
CAGCTATCCATTGGTCTTAGGCCCCAAAAATTTTGGTGCAACTCCAAATAAAAGTAATAACCATGCACACTACT
ATAACCACCCTAACCCTGACTTCCCTAATTCCCCCCATCCTTACCACCCTCGTTAACCCTAACAAAAAAAACTCA
TACCCCCATTATGTAAAATCCATTGTCGCATCCACCTTTATTATCAGTCTCTTCCCCACAACAATATTCATGTG
CCTAGACCAAGAAGTTATTATCTCGAACTGACACTGAGCCACAACCCAAACAACCCAGCTCTCCCTAAGCTTCA
AACTAGACTACTTCTCCATAATATTCATCCCTGTAGCATTGTTCGTTACATGGTCCATCATAGAATTCTCACTG
TGATATATAAACTCAGACCCAAACATTAATCAGTTCTTCAAATATCTACTCATTTTCCTAATTACCATACTAAT
CTTAGTTACCGCTAACAACCTATTCCAACTGTTCATCGGCTGAGAGGGCGTAGGAATTATATCCTTCTTGCTCA
TCAGTTGATGATACGCCCGAGCAGATGCCAACACAGCAGCCATTCAAGCAATCCTATACAACCGTATCGGCGAT
ATCGGTTTCATCCTCGCCTTAGCATGGTTTATCCTACACTCCAACTCATGAGACCCACAACAAATAGCCCTTCT
AAACGCTAATCCAAGCCTCACCCCACTACTAGGCCTCCTCCTAGCAGCAGCAGGCAAATCAGCCCAATTAGGTCT
CCACCCCTGACTCCCCTCAGCCATAGAAGGCCCCACCCCAGTCTCAGCCCTACTCCACTCAAGCACTATAGTTGT
AGCAGGAGTCTTCTTACTCATCCGCTTCCACCCCCTAGCAGAAAATAGCCCACTAATCCAAACTCTAACACTAT
GCTTAGGCGCTATCACCACTCTGTTCGCAGCAGTCTGCGCCCTTACACAAAATGACATCAAAAAAATCGTAGCC
TTCTCCACTTCAAGTCAACTAGGGCTCATAGTAGTTACAATCGGCATCAACCAACCACACCTAGCATTCCTGCA
CATCTGTACCCACGCCTTCTTCAAAGCCATACTATTTATATGCTCCGGGTCCATCATCCACAACCTTAACAATG
AACAAGATATTCGAAAAATAGGAGGACTACTCAAAACCATACCTCTCACTTCAACCTCCCTCACCATTGGCAGC
CTAGCATTAGCAGGAATACCTTTCCTCACAGGTTTCTATTCCAAAGACCACATCATCGAAACCGCAAACATATC
ATACACAAACGCCTGAGCCCTATCTATTACTCTCATCGCTACCTCCCTGACAAGCGCCTATAGCACTCGAATAA
TTCTTCTCACCCTAACAGGTCAACCTCGCTTCCCTACCCTTACTAACATTAACGAAAATAACCCCACCCTACTAA
ACCCCATTAAACGCCTGGCAGCCGGAAGCCTATTCGCAGGATTTCTCATTACTAACAACATTCCCCCCGCATCCC
CCTTCCAAACAACAATCCCCCTCTACCTAAAACTCACAGCCCTCGCTGTCACTTTCCTAGGACTTCTAACAGCCC
TAGACCTCAACTACCTAACCAACAAACTTAAAATAAAATCCCCACTATACACATTTTATTTCTCCAACATACTC
GGATTCTACCCTAGCATCACACACCGCACAATCCCCTATCTAGGCCTTCTTACGAGCCAAAACCTGCCCCTACTC
CTCCTAGACCTAACCTGACTAGAAAAGCTATTACCTAAAACAATTTCACAGCACCAAATCTCCACCTCCATCAT
CACCTCAACCCAAAAAGGCATAATTAAACTTTACTTCCTCTCTTTCTTCTTCCCACTCATCCTAACCCTACTCCT
AATCACATAACCTATTCCCCCGAGCAATCTCAATTACAATATATACACCAACAAACAATGTTCAACCAGTAACT
ACTACTAATCAACGCCCATAATCATACAAAGCCCCCGCACCAATAGGATCCTCCCGAATCAACCCTGACCCCTCT
CCTTCATAGATTATTCAGCTTCCTACACTATTAAAGTTTACCACAACCACCACCCCATCATACTCTTTCACCCAC
AGCACCAATCCTACCTCCATCGCTAACCCCACTAAAACACTCACCAAGACCTCAACCCCTGACCCCCATGCCTCA

GGATACTCCTCAATAGCCATCGCTGTAGTATATCCAAAGACAACCATCATTCCCCCTAAATAAATTAAAAAAAC
TATTAAACCCATATAACCTCCCCCAAAATTCAGAATAATAACACACCCGACCACACCACTAACAATCAATACTA
AGCCCCCATAAATAGGAGAAGGCTTAGAAGAAAACCCCACAAACCCCATTACTAAACCCACACTCAACAGAAAC
AAAGCATACATCATTATTCTCGCACGGACTACAACCACGACCAATGATATGAAAAACCATCGTTGTATTTCAAC
TACAAGAACACCAATGACCCCAATACGCAAAATTAACCCCCTAATAAAATTAATTAACCACTCATTCATCGACC
TCCCCACCCCATCCAACATCTCCGCATGATGAAACTTCGGCTCACTCCTTGGCGCCTGCCTGATCCTCCAAATCA
CCACAGGACTATTCCTAGCCATGCACTACTCACCAGACGCCTCAACCGCCTTTTCATCAATCGCCCACATCACTC
GAGACGTAAATTATGGCTGAATCATCCGCTACCTTCACGCCAATGGCGCCTCAATATTCTTTATCTGCCTCTTC
CTACACATCGGGCGAGGCCTATATTACGGATCATTTCTCTACTCAGAAACCTGAAACATCGGCATTATCCTCCT
GCTTGCAACTATAGCAACAGCCTTCATAGGCTATGTCCTCCCGTGAGGCCAAATATCATTCTGAGGGGCCACAG
TAATTACAAACTTACTATCCGCCATCCCATACATTGGGGCAGACCTAGTTCAATGAATCTGAGGAGGCTACTCA
GTAGACAGTCCCACCCTCACACGATTCTTTACCTTTCACTTCATCTTGCCCTTCATTATTGCAGCCCTAGCAGCA
CTCCACCTCCTATTCTTGCACGAAACGGGATCAAACAACCCCCTAGGAATCACCTCCCATTCCGATAAAATCACC
TTCCACCCTTACTACACAATCAAAGACGCCCTCGGCTTATTTCTCTTCCTTCTCTCCTTAATGACATTAACACTA
TTCTCACCAGACCTCCTAGGCGACCCAGACAATTATACCCTAGCCAACCCCTTAAACACCCCTCCCCACATCAAG
CCCGAATGATATTTCCTATTCGCCTACACAATTCTCCGATCCGTCCCTAACAAACTAGGAGGCGTCCTTGCCCTA
TTACTATCCAGTCCTCATCCTAGCAATAATCCCCATCCTCCATATATCCAAACAACAAAGCATAATATTTCGCC
CACTAAGCCAATCACTTTATTGACTCCTAGCCGCAGACCTCCTCATTCTAACCTGAATCGGAGGACAACCAGTA
AGCTACCCTTTTATCATCATTGGACAAGTAGCATCCGTACTATACTTCACAACAATCCTAATCCTAATACCAAC
TATCTCCCTAATTGAAAACAAAATACTCAAATGGACCTGTCCTTGTAGTATAAACTAATACACCAGTCTTGTAA
GCCGGAGATGAAAACCTTTTTCCAAGGACAAATCAGAGAAAAAGTCTTTAACTCCACCATTAGCACCCAAAGCT
AAGATTCTAATTTAAACTATTCTCTGTTCTTTCATGGGGAAGCAGATTTGGGTACCACCCAAGTATTGACTCAC
CCATCAACAACCGCTATGTATTTCGTACATTACTGCCAGACACCATGAATATTGTACAGTACCATAAATACTTG
ACCACCTATAGTACATAAAAACCCAATCCACATCAAAACCCTCCCCCCATGCTTACAAGCAAGTACAGCAATCA
ACCTTCAACTGTCATACATCAACTGCAACCCCAAAGCCACCCCTCCCCCACTAGGATACCAACAAACCTACTCAC
CCTTAACAGTACATAGCACATAAAGCCATTTACCGTACATAGCACATTACAGTCAAATCCCTTCTCGTCCCCAT
GGATGACCCCCCTCAGATAGGGGTCCCTTGACCACCATCCTCCGTGAAATCAATATCCCGCACAAGAGTGCTAC
TCTCCTCGCTCCGGGCCCATAATACTT

>Pse_14
GCTAAGAACTTCATTGGTTTCCTAGGGTTTATTGTGTGAGCACACCATATATTTACAGTAGGAATGGACGTAG
ACACACGAGCTTATTTCACCTCCGCTACCATAATCATCGCTATCCCCACCGGCGTCAAAGTATTTAGCTGACTCG
CTACACTCCACGGAAGCAATATGAAGTGATCTGCTGCAGTGCTCTGAGCCCTGGGATTCATTTTTCTCTTCACT
GTGGGTGGCCTAACCGGCATTGTATTAGCAAACTCATCACTAGACATCGTACTACACTACAC

>Pse_15
GATTACACATGCAAGCATCCCCGTCCCGGTGAGTCACCCTCTAAATCACCACGATCAAAAGGAACAAGTATCAA
GCACGCAGCAATGCAGCTCAAAACGCTTAGCCTAGCCACACCCCCATGGGAGACAGCAGTGATAAACCTTTAGC
AATAAACGAAAGTTTAACTAAGCTATACTAACTCCAGGGTTGGTCAATTTCGTGCCAGCCACCGCGGTCACACG
ATTAACCCAAGTCAATAGAAACCGGCGTAAAGAGTGTTTTAGAT

>Pse_16

CATTAACCTTTTAAGTTAAAGATTAAGAGAACCAACACCTCTTTACAGTGAAATGCCCCAACTAAATACTACCG
TATGGCCCACCATAATTGCCCCCATACTTCTTACACTATTCCTCATCACTCAGCTAAAAATATTAAATACAAAT
TACCATCTACCCCCCTCACCAAAGCCCATAAAAATAAAAAACTATAGTAAACCCTGAGAACCAAAGTGAACGAA
AATCTGTTCGCTTCATTCATTGCCCCCACAATCCTAGGTCTGCCCGCCGCAGTACTGATCATTCTATTTCCCCCT
CTATTGATCCCCACCTCCAAATACCTCATCAACAACCGACTAATTACCACCCAACAATGACTAATCCAACTAACC
TCAAAACAAATGATAGCCATACACAACACTAAAGGGCGAACCTGATCTCTTATACTAGTATCCTTAATCATTTT
TATTGCCACAACTAATCTTCTCGGGCTCCT
>Pse_17
GGGGCCCTCTCAGCCCTCCTAATGACCTCCGGCCTAGCCATGTGATTCCACTTCCACTCCACAACCCTACTCATA
CTAGGCCTACTAACCAACACACTAACCATATACCAATGATGGCGCGATGTAACACGAGAAAGCAC

>Pse_19
CAACATGGCTTTCTCAACTTTTAAAGGATAACAGCCATCCGTTGGTCTTAGGCCCCAAAAATTTTGGTGCAACT
CCAAATAAAAGTAATAACCATGTATGCCACTATAACCACCCTAGCCCTAACTTCCTTAATTCCCCCCATCCTTAC
CACCTTCATTAACCCTAACAAAAAAA

>Pse_21
ATAAAATTAATTAACCACTCATTTATCGACCTCCCCACCCCATCCAACATCTCCGCATGATGAAACTTCGGCTC
ACTCCTTGGCGCCTGCCTGATCCTCCAAATCACCACAGGACTATTCCTAGCCATGCACTACTCACCAGAYGCCTC
AACCGCCTTTTCATCAATCGCCCATATCACCCGAGACGTAAATCATGGCTGAATCATCCGCTACCTTCACGCCAA
TGGCGCCTCAATATTCTTTATC

>Pse_22
TCGCCCACTAAGCCAATCACTTTATTGACTCCTAGCCGCAGACCTCCTCATTCTAACCTGAATCGGAGGACAACC
AGTAAGCTACCCTTTTATCATCATTGGACAAGTAGCATCCGTACTATACTTCACAACAATCCTAATCCTAATAC
CAACTATCTCCCTAATTGAAAACAAAATACTCAAATGGGCCTGTCCTTGTAGTATAAACTAATACACCAGTCTT
GTAAACCGGAGATGAAAACCTTTTTCCAAGGACAAATCAGAGAAAA
